# RNA modification mapping with JACUSA2

**DOI:** 10.1186/s13059-022-02676-0

**Published:** 2022-05-16

**Authors:** Michael Piechotta, Isabel S. Naarmann-de Vries, Qi Wang, Janine Altmüller, Christoph Dieterich

**Affiliations:** 1 Klaus Tschira Institute for Integrative Computational Cardiology, University Hospital Heidelberg, Im Neuenheimer Feld 669, Heidelberg, 69120 Germany; 2 German Center for Cardiovascular Research (DZHK), Partner site Heidelberg/Mannheim, Heidelberg, 69120 Germany; 3 Cologne Center for Genomics (CCG), Weyertal 115b, Cologne, 50931 Germany

**Keywords:** m6A, Pseudouridine, Reverse transcription signature, Nanopore

## Abstract

Several high-throughput antibody-free methods for RNA modification detection from sequencing data have been developed. We present JACUSA2 as a versatile software solution and comprehensive analysis framework for RNA modification detection assays that are based on either the Illumina or Nanopore platform. Importantly, JACUSA2 can integrate information from multiple experiments, such as replicates and different conditions, and different library types, such as first- or second-strand cDNA libraries. We demonstrate its utility, showing analysis workflows for N6-methyladenosine (m6A) and pseudouridine (*Ψ*) detection on Illumina and Nanopore sequencing data sets. Our software and its R helper package are available as open source solutions.

**Supplementary Information:**

The online version contains supplementary material available at (10.1186/s13059-022-02676-0).

## Background

Lately, we have seen a whole wave of antibody-free methods to map RNA modifications transcriptome-wide based on high-throughput sequencing data [1, 2]. What is common to all of these approaches is that, independent of the actual protocol, RNA modification sites manifest as specific read signatures in sequencing data. Others and we have previously worked on mapping modifications, which originate from RNA editing [3]. For example, adenosine deamination is a process that typically converts A → I and is facilitated by the family of ADAR proteins [4]. Previously, we had contributed JACUSA [5] to this exciting field of RNA modification research.

In this manuscript, we present the successor JACUSA2 (https://github.com/dieterich-lab/JACUSA2), which features better running time performance and captures more complex read signatures involving base substitutions, insertions, deletions, and read truncations (see Additional file 1: Fig. S1A-C). Oftentimes, these signatures are introduced at the reverse transcription step (RNA → cDNA, so called reverse transcription signature). Novel antibody-free approaches have been established for the mapping of N6-methyladenosine (m6A), 5-methylcytosine (m5C), pseudouridine (*Ψ*), and N1-methyladenosine (m1A) and beyond by sequencing (reviewed in [6]). In this manuscript, we focus on messenger RNA (mRNA) modifications in general and N6-methyladenosine (m6A) in particular. However, our software approach generalizes to other RNA species (e.g. human rRNA) and all of the aforementioned modifications as the presence of a modified residue typically manifests as a site-specific increase in 5’ or 3’ read termination, base misincorporation, and insertion or deletion rate [7]. An additional feature is the possibility to stratify reads by a particular base substitution, which could occur anywhere on the read. JACUSA2 is able to process data from any sequencing platform as long as it can be provided in the binary sequence alignment map format (BAM), a universal standard format [8]. In the following, we have applied JACUSA2 to sequencing data from the Illumina and ONT Nanopore platform across multiple use cases. Downstream analyses of JACUSA2 output are supported by an additional R package JACUSA2helper (https://github.com/dieterich-lab/JACUSA2helper) Our manuscript is accompanied by Supplementary Material (see Additional file 1), which provides detailed information on methods, benchmarks and performance.

## Results and discussion

The first use case focuses on a methylation-sensitive RNA restriction enzyme (MazF) assay to identify m6A modified residues in mRNA of HEK293 cells [9, 10]. The second use case complements the first by demonstrating a genetic approach to map m6A sites in HEK293 cells using DART-seq [11]. While all of the aforementioned use cases utilized Illumina sequencing data, the third use case analyzes Nanopore direct mRNA sequencing data from HEK293 wild-type and knockout cells (*METTL3* -/-) [12].

### Use case 1: m6A mapping by MazF treatment and sequencing

**Fig. 1:**
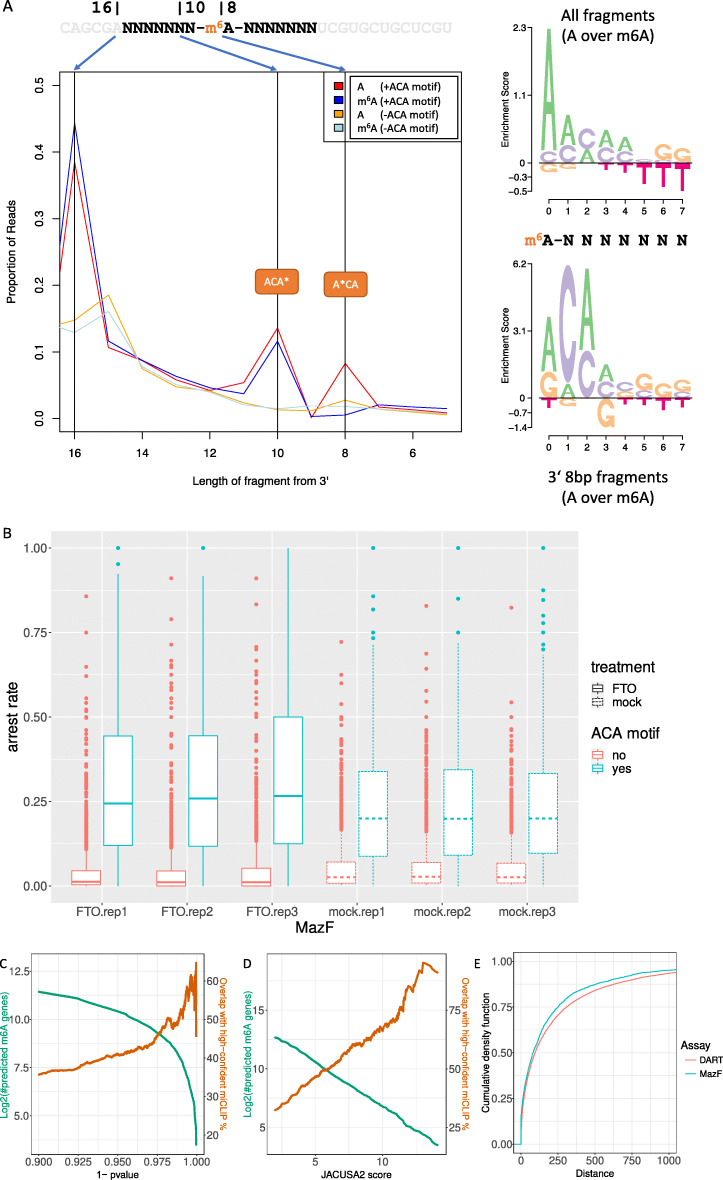
Summary of JACUSA2 results on MazF restriction enzyme assay and DARTseq assay in HEK293 cells. **A** Synthetic RNA Oligo digestion and sequencing. Position 8 hosts the central A/m6A. Position 1-7 and 9-16 are random. We have sequenced 2 libraries, which differ in position 8 (A/m6A). The MazF recognition motif becomes apparent when we consider cleaved fragments (3 ′ 8bp fragments). **B** Arrest rate analysis across replicates in published data from HEK-293 cells [9]. Results are shown for a set of 2792 well covered sites with robust and significant read arrest changes. One-sided Wilcoxon signed-rank test: Maz-FTO-ACA vs. MAZ-mock-ACA; one-sided Wilcoxon rank sum test pvalue <10^−16^. **C** Comparison of JACUSA2 *rt-arrest* predictions for [9] to a high-confident set of miCLIP sites (intersection of 3 miCLIP experiments [15–17]) on gene set level. **D** Comparison of JACUSA2 *call-2* predictions for [11] to a high-confident set of miCLIP sites (intersection of 3 miCLIP experiments [15–17]) on gene set level. **E** cDNA distance of closest miCLIP site (intersection of 3 miCLIP experiments) to the respective MazF cleavage site (red) or DART C →U site (turquoise). Wilcoxon rank sum test pvalue: 5.957*e*−13

Lately, we have seen a whole wave of antibody-free methods to map m6A RNA modifications transcriptome-wide based on high-throughput sequencing data. A need for antibody-free complementary approaches has been clearly identified by a rigorous assessment of published MeRIP data sets. McIntyre et al. show that m6A peak overlap varies from ≈30 to 60% between studies, even in the same cell type [13]. An antibody-independent solution to map m6A sites is provided by an alternative biochemical approach, namely m6A methylation-sensitive enzymatic RNA digestions [9, 10]. We have tested the substrate specificity of the MazF enzyme on custom RNA oligos with a centrally modified A position. Figure 1A gives an overview on the taken approach to assess MazF substrate specificity. Briefly, the central A/m6A position is flanked by 7 random nucleotides on each site and a constant prefix and suffix. Total sequence length per oligo is 34bp. These RNA oligos were subjected to enzymatic digestion. As previously reported, MazF cuts 5’ to an ACA motif. Cutting efficiency is reduced by the presence of an m6A modification (A*CA motif, asterisk denotes modification). Importantly, the enzymatic reaction does not differ for cutting 5’ of ACA or ACA*, but only differs in the case of A*CA. We have reanalyzed the 6 Illumina libraries from Zhang et al. [9] using the new mode *rt-arrest*. Briefly, 3 replicates were treated with an Alpha-ketoglutarate-dependent dioxygenase (FTO), which converts m6A to A on mRNA [14], and 3 replicates were mock-treated. MazF cleavage efficiency (or arrest rate) does increase in the absence of m6A. Figure 1B shows the observed arrest rates in the data stratified by the presence or absence of an ACA motif. Arrest rates are significantly elevated in FTO-treated samples as compared to mock-treated samples. This characteristic read signature is used to predict m6A modified sites in mRNA in a head-to-head comparison using JACUSA2 (FTO vs mock treated samples). Figure 1C shows the number of predicted m6A gene targets (≥1 predicted site) and their respective overlap with consensus miCLIP gene targets, which are shared 3 independent experiments [15–17] depending on the *p*−*value* from the beta-binomial test in JACUSA2 *rt-arrest*. Our R package JACUSA2helper provides a vignette for MazF assay data analysis. Finally, a comparison to the MAZTER-mine workflow [10] can be found in the Additional file 1 on page 5. Briefly, JACUSA2 outperforms MAZTER-mine in terms of running time by orders of magnitude.

### Use case 2: DART-seq

A complementary genetic approach is an extension of the TRIBE technique called DART-seq [11]. Meyer applied DART-seq on HEK293 cells where the APOBEC domain was fused to the YTH domain from human YTHDF2 (WT and mutated). In essence, new C →U editing events that are significantly enriched in the YTHDF2-WT, but not in the binding domain mutant are *bona fide* candidates for m6A RNA modification. Elevated C → U deamination on RNA level for the active domain over the mutated domain are used to predict m6A modifications on mRNA. Figure 1D shows the number of predicted m6A target genes (≥1 predicted site) and their respective overlap with consensus miCLIP gene targets as explained before. Generally, DART-seq has the advantage that it can be extended to 3rd generation long read sequences and requires only very little RNA input amounts (in vivo experiments) yet relies on genetic modification of the target system / organism. Additional file 1: Fig. S3 summarizes the analysis results as Venn diagram. Figure 1E shows the cDNA distance of either a MazF or a DART-seq target site to its closest miCLIP site. The majority of detected sites is less than 200bp away from the closest miCLIP peak. Our R package JACUSA2helper provides a vignette for DARTseq assay data analysis. Additional file 1: Fig. S4 summarizes the agreement between miCLIP, DART-seq and MazF experiments.

### Use case 3: Nanopore direct mRNA sequencing

**Fig. 2:**
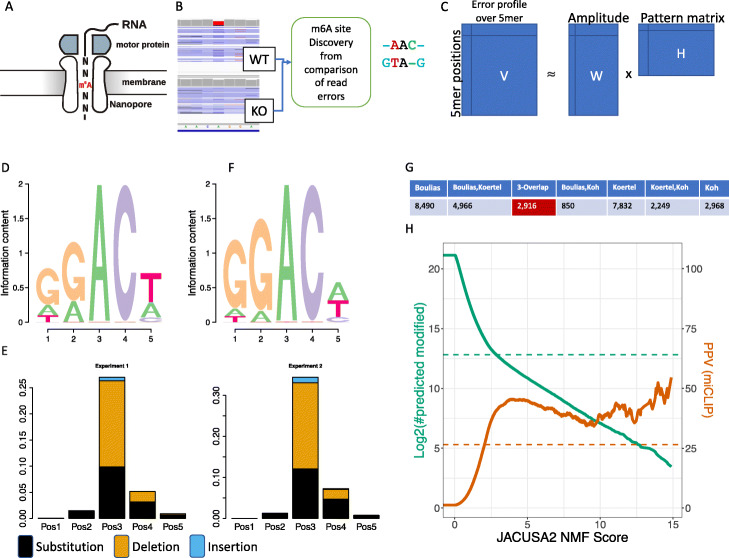
Nanopore sequencing of HEK293 cell lines: Mettl3 KO and WT. **A** Direct RNA sequencing on Nanopore. **B** Signature detection with JACUSA2 comparing WT over KO cells. Three principal events are detected: base substitutions, insertions and deletions. All 5mers with a central A (NNANN) are considered. **C** Non-negative matrix factorization to identify characteristic patterns (matrix H) that are indicative of the m6A modification. **D** Sequence logo of miCLIP training data (2916 sites (5mers), red 3-overlap in **G**). **E** Profile of the strongest signal from the NMF pattern matrix (H, 4th row aka NMF4) across two independent Nanopore experiments. **F** Sequence enrichment logo for sites with Score(NMF4 >>other patterns). G: miCLIP subsets: red 3-overlap was used for NMF training, others (shown in light blue) are used for testing. H: JACUSA2 m6A predictions: total number of predicted m6A sites in green and exact overlap with miCLIP site in brown (test data, 27,355 sites). PPV = #true CLIP sites / (#true + #false predictions). xPore performance is indicated with dashed lines

Direct RNA sequencing without involving any cDNA library preparation or PCR-based amplification steps is a unique feature of the ONT sequencing platform. Briefly, native poly-adenylated RNA is threaded through a nanopore in 3’ → 5’ orientation (Fig. 2A). Some ground-breaking work on m6A detection by direct RNA sequencing has been performed by Liu et al. [18] in yeast and Jenjaroenpun et al. [7] in mouse embryonic stem cells. We have identified a novel published direct RNA sequencing data set from HEK293 cells [12], which facilitates a direct comparison to the aforementioned Illumina-based approaches. Moreover, there are 3 miCLIP data sets available for HEK293 cells [15–17], which we use as an antibody-based reference set.

We applied JACUSA2 (enhanced *call-2* mode) to identify read error profile differences between the respective paired samples (see Fig. 2B). We are considering all positions with sufficient read coverage (> 4) across all replicates and focus on positions with a specific 5mer context, which is basically a central A nucleotide flanked by 2 adjacent random nucleotides (NNANN). Our initial unsupervised learning approach (Non-negative matrix factorization, NMF) is shown in Fig. 2C. We use 2916 previously reported consensus miCLIP m6A sites from [15–17] as training set to identify characteristic patterns. The sequence composition of the 5-mer training data is shown as sequence logo in Fig. 2D.

Only non-overlapping 5mers outside of homo-polymer regions (JACUSA filter: Y) are subsequently used in training. The respective feature set consists of deletion, insertion, and substitution scores for every considered 5mer. This amounts to a feature matrix of 3 event scores ×5 motif positions ×2 experiments = 30 features. We subjected this feature matrix to a non-negative matrix factorization (NMF) analysis (Fig. 2C). Through robustness and concordance assessment, we could identify an optimal factorization rank of 5. The pattern matrix (H) highlights the importance of pos 3 (and 4) for the most relevant pattern (Fig. 2E). The loading of this factor correlates with the presence of the well known DRACH motif and can be reproduced across two independent experiments in HEK293 cells (Fig. 2D, data from [12]). The most relevant pattern is now applied to previously unseen miCLIP-predicted m6A sites (Fig. 2G). Figure 2G uses all light blue miCLIP sets as ground truth annotation and shows the overlap of JACUSA2 predictions with the corresponding miCLIP sets as well as the total number of predictions. The published performance of xPore is denoted with dashed lines (taken from SData 1 in [12]). A comparative assessment of JACUSA2 with Epinano 1.2 [18], ELIGOS2 [7] and xPore [12] is presented as part of the text in Additional file 1 on page 6ff. In short, only xPore produced competitive results in a direct comparison with JACUSA2 (see also Additional file 1: Fig. S5). Of note, the learned m6A pattern in Fig. 2E is represented by the sequence context in Fig. 2F and can be transferred to other species and cell types. To demonstrate this transfer, we use a murine model system, mouse embryonic stem cells (mESC). Jenjaroenpun et al. [7] has sequenced mESC mRNA on the Nanopore and Köertel et al. [16] mapped m6A residues via a novel miCLIP approach (antibody-based reference set). Additional file 1: Fig. S6A describes the covered adenosine residues of interest (NNANN) and their overlap with the miCLIP m6A sites. Additional file 1: Fig. S6B describes the concept and shows the top scoring predicted m6A sites from the Nanopore data, which are all supported by miCLIP-derived predictions. The global score distribution for mouse predictions is indistinguishable from CLIP-supported human prediction scores (Additional file 1: Fig. S6C).

### Use case 4: Nanopore direct rRNA sequencing

**Fig. 3:**
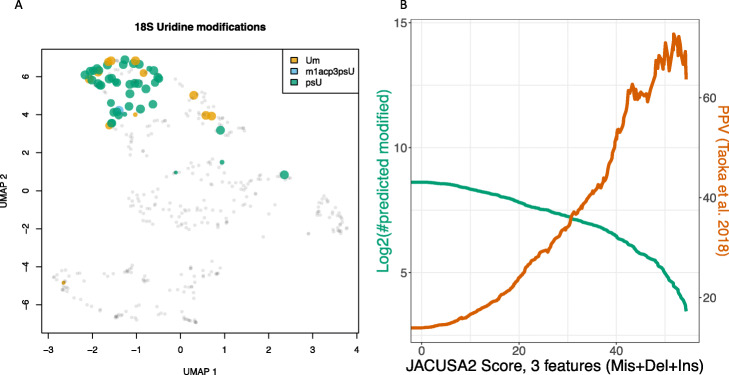
Nanopore sequencing of human 18S rRNA from HEK293 cells. **A** UMAP projection of JACUSA2 base substitution, insertion, and deletion scores for uridine positions. psU stands for *Ψ* modified positions, Um stands for 2 ′-O-methyl ribose modifications and m1acp3psU stands for 1-methyl-3-(3-amino-3-carboxypropyl)pseudouridine-5 ′-monophosphate. **B** Line plot showing by ascending sum of JACUSA2 *call-2* scores the number of predictions in green and the overlap with annotated uridine modifications in brown. Modified positions as defined by Taoka et al. [19]

Complementarily, we show case the utility of JACUSA2 on another RNA species: human 18S rRNA. We have established custom adapters for rRNA sequencing (Naarmann-de Vries et al. in preparation) and sequenced both, HEK293 wild-type 18S rRNA molecules and unmodified in vitro transcribed 18S rRNA molecules. In this particular use case, we employ JACUSA2 to identify uridine modifications. Taoka et al. [19] define the human rRNA modification landscape and estimated modification levels. We use this information to validate JACUSA2 scores for uridine modification predictions. Briefly, 42 *Ψ* and 12 2’-O-methyl uridines have been mapped on human 18S rRNA along with 339 unmodified uridines. We compute single nucleotide base substitution, deletion and insertion scores with JACUSA2 for every U residue. The UMAP projection of these scores is shown in Fig. 3A. A distinct “cluster” of modified residues exists, which is well separated from the rest of uridine residues in this respective projection. The size and color of the dots represent annotated modification levels and modification type, respectively. Figure 3B summarizes the performance of JACUSA2 by showing the number of predictions based on JACUSA2 *call-2* scores in green and the overlap with annotated uridine modifications in brown. A comparison to a very recent solution for pseudouridine prediction, NanoPsu [20], is summarized in Additional file 1 on page 10 (see also Additional file 1: Fig. S7). Taken together JACUSA2 is able to predict uridine modifications using Nanopore sequencing data. An orthogonal show case using conventional Illumina sequencing employs a chemical approach to modify pseudouridines with carbodiimide and was analyzed with JACUSA2 as well (see Additional file 1 - Additional Use Case: Pseudouridylation site mapping in human rRNAs and Additional file 1: Fig. S8).

## Conclusion

We present JACUSA2 as a versatile software solution and comprehensive analysis framework for RNA modification detection assays that are based on either the Illumina or Nanopore platform. We have explored different use cases for m6A and uridine modification detection. All of which use antibody-free methods and encompass complex read signatures. JACUSA2 supersedes our initial software in terms of features and speed (see Additional file 1: Fig. S1+2). Result tables for all presented use cases are available as Additional file 2, 3, 4, 5, and 6. JACUSA2 is complemented with an R package (JACUSA2helper), which eases downstream processing of JACUSA2 output (https://github.com/dieterich-lab/JACUSA2helper). We provide all relevant information to reproduce our results from the presented workflows as vignettes in the JACUSA2helper repository (https://dieterich-lab.github.io/JACUSA2helper/). We will continue to develop JACUSA2 to meet new requirements in terms of prediction performance for all aforementioned sequencing platforms.

## Methods

### Variant calling

The existing variant calling framework in JACUSA1 has been reimplemented for JACUSA2 and variant calling for only one condition has been added. In this single condition variant calling mode, an in silico condition is created from the sequencing data by transforming non-reference bases to conform with the reference sequence. A reference FASTA can be provided by the user or the MD-field from the SAM File Format is used to reconstruct the reference sequence.

### General modelling framework

In order to model counts beyond base calls observed in variant calling, we revised the original implementation of the Dirichlet-Multinomial distribution ***x***∼*DirMult*(*α*_1_,…,*α*_*K*_) from JACUSA1. The goal was to model category count vectors ***x***=(*x*_1_,…,*x*_*K*_) for arbitrary *K*.

In variant variant calling, the category count vector ***x*** consist of 4 elements and is defined as the observed base call counts ***x***_***BC***_=(*x*_*A*_,*x*_*C*_,*x*_*G*_,*x*_*T*_) at an arbitrary site within the genome. The Dirichlet Multinomial *DirMult*(***α***) is used to model base call counts ***x***_***BC***_ and the Newton iteration method presented by Minka (https://tminka.github.io/papers/minka-newton.pdf) is used to determine parameters ***α***.

We implemented a general framework based on the Beta-Binomial distribution to model counts of binary features derived from reads. The Beta-Binomial distribution *BetaBin*(*α*,*β*) can be defined as a special case of the more general *DirMult*(*α*_1_,…,*α*_*K*_) when *K*=2. Given some arbitrary binary feature *y* the count vector is defined as ***y***=(*y*_1_,*y*_2_):*y*=*y*_1_+*y*_2_. To account for zero counts and where appropriate, we add a pseudocount of one.

For each observed and pooled count vectors *BetaBin*(*α*,*β*) distributions are fitted with the aforementioned method and compared with a likelihood ratio test. The null hypothesis is that the counts of each condition (*c*∈*I*,*II*) have the same underlying distribution:

$$LL=-2 \cdot \log \frac{BetaBin(\alpha^{I}, \beta^{I}) \cdot BetaBin(\alpha^{II}, \beta^{II})}{BetaBin(\alpha^{I + II}, \beta^{I + II})} $$

We approximate *LL* with a *χ*^2^ distribution and provide the *p*-value.

#### Modelling read arrest events

An arrest event is identified by comparing stop and read through events counts between two conditions (*c*∈{*I*,*II*}). Given the general modelling framework, the category count vector is defined as the number of observed *A*rrest events and read *T*hrough events *y*_*AT*_=(*y*_*arrest*_,*y*_*through*_).

The arrest rate *r* is defined as the fraction of arrest reads and total reads:

$$r_{arrest} = \frac{y_{arrest}}{y_{arrest} + y_{through}} $$

We model ***y***_***AT***_ with the Beta-Binomial distribution and use the aforementioned general framework to perform a statistical test. We report the test-statistic and the approximated *p*-value for each candidate arrest site.

#### Modelling INDELs

The underlying statistical framework of INDEL calling is comparable to the previously presented modelling of read arrest events. The general modelling framework is used to model observed insertion and/or deletion counts. In insertion calling, the category vector $\boldsymbol {y_{I\overline {I}}}$ is defined as the number of observed reads with (*y*_*ins*_) and without ($y_{\overline {ins}}$) an insertion: $\boldsymbol {y_{I\overline {I}}} = (y_{ins}, y_{\overline {ins}}) : y_{\overline {ins}} = y_{tot} - y_{ins}$. The total number of reads (*y*_*tot*_) includes all reads that span the respective insertion, including intron regions and low quality base calls. For each insertion site the category vector $\boldsymbol {y_{I\overline {I}}}$ is modelled with the Beta-Binomial distribution. The test-statistic and the approximated *p*value for each insertion site are reported.

Deletion calling works analogously to insertion calling with the category vector defined as: $\boldsymbol {y}_{D\overline {D}} = (y_{del}, y_{\overline {del}})$. Similar modelling and reporting applies here.

## Supplementary Information

**Additional file 1** Supplementary Text (PDF). Additional sures and results.

**Additional file 2** Table S1 (XLSX). m6A target site predictions from MazF-FTO differential digestion assay.

**Additional file 3** Table S2 (XLSX). m6A target site predictions from YTH-APOBEC mapping assay.

**Additional file 4** Table S3 (XLSX). m6A target site predictions from Nanopore direct RNA-seq (KO vs. WT).

**Additional file 5** Table S4 (XLSX). *Ψ* target site predictions from CMC *rt-arrest* assay [21].

**Additional file 6** Table S5 (XLSX). *Ψ* target site predictions from Nanopore direct human 18S rRNA sequencing.

**Additional file 7** Review history (DOCX). Peer review history.

## Data Availability

A comprehensive list of all sequencing data sets can be found in Supplementary Materials. Our software is distributed under GPL-v3 and can be either found at the JACUSA2 GitHub repository (JAR files + tutorials, https://github.com/dieterich-lab/JACUSA2), the JACUSA2helper GitHub repository (R package + vignettes, https://github.com/dieterich-lab/JACUSA2helper), or at [22]. BAM files and JACUSA2 output files can be retrieved from Zenodo 10.5281/zenodo.5930728. Our human 18S rRNA sequencing data are available on NCBI SRA through https://www.ncbi.nlm.nih.gov/bioproject/824272. We used the following published data sets for our analyses: *Use Case 1: m6A mapping by MazF treatment and sequencing* Zhang, Zhang et al. [9]. Single base mapping of m6A by an antibody-independent method. NCBI Short Read Archive (SRP179955) https://www.ncbi.nlm.nih.gov/sra?term=SRP179955 Runs: SRR8450805, SRR8450807, SRR8450809, SRR8450806, SRR8450808, SRR8450810 *Use Case 2: DART-seq*: Meyer, Kate [11]. An antibody-free method for global m6A detection. NCBI Short Read Archive (SRP182709) https://www.ncbi.nlm.nih.gov/sra?term=SRP182709 Runs: SRR9940470, SRR9940471, SRR9940472, SRR9940474, SRR9940475, SRR9940476 *Use Case 3: Nanopore direct mRNA sequencing*: Pratanwanich, P.N. [12]. Detection of differential RNA modifications from direct RNA sequencing of human cell lines. NCBI Short Read Archive (ERP124567) https://www.ncbi.nlm.nih.gov/sra?term=ERP124567 Runs: ERR4706162, ERR4706163, ERR4706159, ERR4706160, ERR4972055, ERR4972057, ERR4972056, ERR4973569 Jenjaroenpun, P. [7], Decoding Epitranscriptional Landscapes form Native RNA Sequences. NCBI Short Read Archive (SRP166020) https://www.ncbi.nlm.nih.gov/sra/?term=SRP166020 Runs: SRR11550232, SRR11550233, SRR11550260, SRR11550261 *Additional Use Case / Supplement: Pseudouridylation site mapping in human rRNAs:* Zhou, K.I. [21]. Pseudouridines have context-dependent mutation and stop rates in high-throughput sequencing. NCBI Short Read Archive (SRP132322) https://www.ncbi.nlm.nih.gov/sra?term=SRP132322.
