# Supplementary material for: RNA modification mapping with JACUSA2
Source: Genome Biol. 2022 May 16;23:115. doi: 10.1186/s13059-022-02676-0 (PMC9109409; doi:10.1186/s13059-022-02676-0)
Supplement: Supplementary file 1 — Additional file 1 Supplementary Text (PDF). Additional sures and results. [file 13059_2022_2676_MOESM1_ESM.pdf]

Supplementary Material

Piechotta Michael
Isabel Naarmann-de Vries
Qi Wang
Janine Altmüller
Dieterich Christoph

May 2, 2022

1 JACUSA2

The variant calling framework from JACUSA1 has been redesigned to enable efficient read processing beyond the identification of single nucleotide variants (SNVs). Native support for single and paired end reads for common stranded and unstranded library types has been added and the core BAM processing component has been upgraded to htslib (`https://github.com/samtools/htsjdk`). A new analysis mode (*rt-arrest*) has been added to JACUSA2 that allows to study read arrest events in paired samples. This works even for combining different library types (see Figure S1B). Furthermore, JACUSA2 allows to identify differential insertion and/or deletion sites. Finally, variant stratification has been added as an optional feature to study the co-occurence of a specific variant and other features such as variants, arrest sites, or INDELs (see Figure S1C). JACUSA2helper (`https://github.com/dieterich-lab/JACUSA2helper`) complements JACUSA2 to study RNA modifications. It is a R package that simplifies downstream analysis and visualization of JACUAS2 output.

We designed a benchmark to compare the running time performance between different implementations based in the variant calling setup (check Figure S2 for detailed results).

General analysis workflow

In general, JACUSA2 features a two tier analysis:

JACUSA2 (Java) fast and accurate variant or arrest event detection and

JACUSA2helper (R) detailed followup analysis and visualization of results.

Given mapped reads as input (BAM files) and corresponding library type information, JACUSA2 will assign aligned reads to separate sequence coordinate windows. Each window is handled by separate threads (i.e. independent set of reads), which enables fast parallel computation on modern machines. Reads are filtered according to user provided parameters, and processed according to the chosen method.

JACUSA2 supports the following main analysis methods:

- simple pileup (*pileup*) of mapped reads from arbitrary number of conditions
- variant calling (*call-{1,2}*) with one or two conditions
- read arrest detection (*rt-arrest*) with two condition

Each method features a statistical test that provides a test-statistic to filter significant candidates. Additionally, each analysis method can be extended to identify differential INDELs and/or to stratify observations based on a specific co-occurring variant.

Optional artifact/feature filtering can be carried out to remove artifacts that cannot be reliably captured by the statistical model (e.g., homopolymers). Finally, results from each threaded window are collected and combined into the final output.

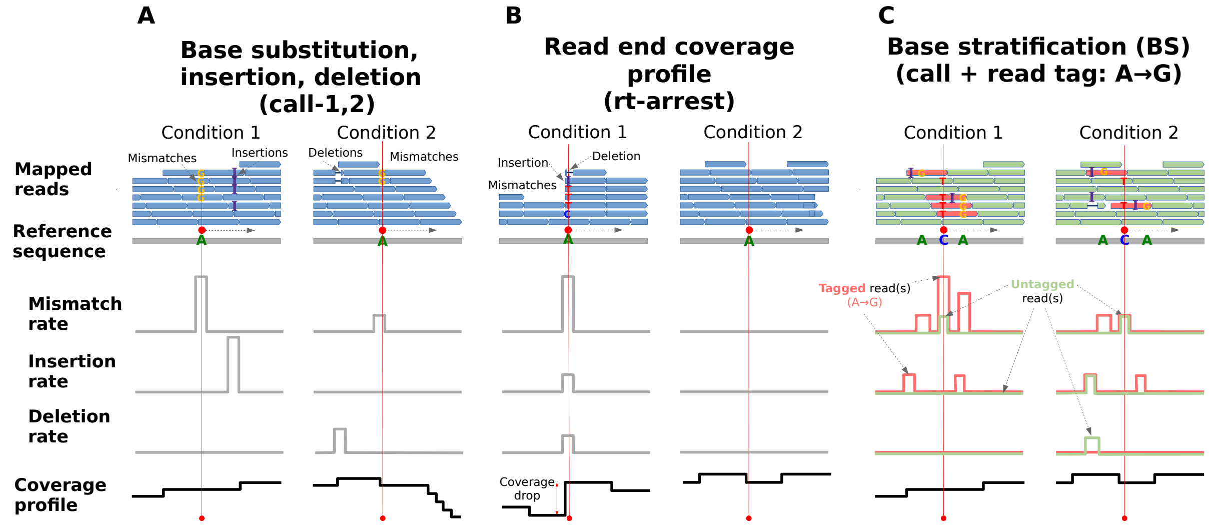

Figure S1: Summary of JACUSA2 analysis options and illustration of utilized sequencing data properties. All analysis options can be extended with INDEL calling where insertion and/or deletions rates between 2 conditions are modelled to identify sites with statistically significant divergent INDEL rates. The matching JACUSA2 command line option is given in parenthesis, e.g.: (call) for variant calling. A: Variant calling: Modelling and comparison of mismatch rates between 1 or 2 conditions. B: Arrest site detection: Coverage profiles of two conditions are modelled to discover statistically significant coverage drops that are termed arrest site. C: Base stratification: Variant calling can be extended with base stratification where reads are grouped or tagged by a specific base substitution. This enables to compare tagged and untagged reads between conditions

Variant stratification

In JACUSA2, a user customizable base substitution can be set to split reads into two categories. Reads that contain the base substitution of interest are termed tagged and the remaining set of reads is called untagged. This variant stratification enables to study the co-occurrence of a variant and some other feature such as arrest events, other variants, or INDELs (see Figure S1C). Variant stratification requires stranded library types and supports single and paired end reads. In paired end reads, a read is termed tagged, if either of the two reads in the pair contains the variant.

Comparison with JACUSA1.*

In brief, the benchmark aims to explore the influence of sequence library type (stranded vs. unstranded) and the number of utilized parallel threads on the running time. We used BAM files restricted to chr1 and chr2 from ADAR knockdown and untreated conditions from [1] as test data to identify varaints between both conditions. On average, the improved JACUSA2 implementation outperforms its predecessors in both unstranded and the more demanding stranded library type setting. More importantly, JACUSA2 scales much better showing a steady decrease of running time when the number of additional CPU cores is increased. In contrast, increasing the number of threads beyond 4 does not show any running time gains for JACUSA1.x. Utilizing 16 cores JACUSA2 completes the unstranded task on average after $\approx$ 500 seconds compared to

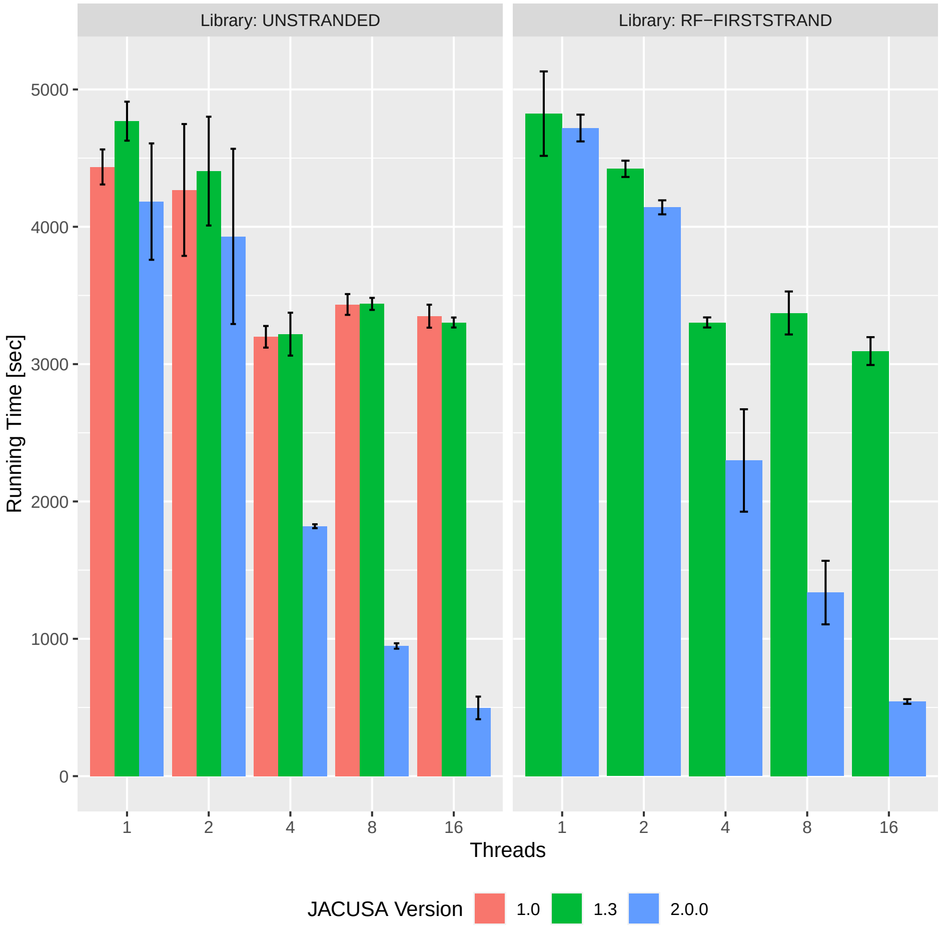

Figure S2: Average running time comparison of call-2 module for different JACUSA versions with unstranded vs. stranded library type (RF-FIRSTSTRAND) and increasing number of used CPU cores (threads). Tested on reduced BAM files (chr1 and chr2) from ADAR knockdown and untreated conditions from [1]. Each run has been repeated 3× (whiskers indicate fastest and slowest running time).

≈ 3.300 seconds for the previous versions. Overall, a significant running time enhancement can be observed when comparing against previous JACUSA versions (see Figure S2).

JACUSA 1.0

JACUSA 1.0 was downloaded and built from the "paper" branch: `https://github.com/dieterich-lab/JACUSA/tree/paper`. JACUSA 1.0 does not support stranded library types, therefore we have only evaluated performance in the unstranded scenario.

The following line was executed to run JACUSA 1.0:

```
$ java -jar JACUSA_v1.0.jar call-2 $OPTS -r $OUTPUT $INPUT
```

JACUSA 1.3

JACUSA 1.3 was downloaded from: `https://github.com/dieterich-lab/JACUSA/releases/download/1.3.0/JACUSA_v1.3.0.jar`.

The following line was executed to run JACUSA 1.3:

```
$java -jar JACUSA_v1.3.0.jar call-2 -P $LIB,$LIB $OPTS -r $OUTPUT $INPUT
```

JACUSA 2

JACUSA 2 was downloaded from: `https://github.com/dieterich-lab/JACUSA2/releases/tag/v2.0.0`.

```
$ java -jar JACUSA_v2.0.0.jar call-2 $OPTS -r $OUTPUT $INPUT"
```

Benchmarking JACUSA2 against other published software

Illumina MazF protocol - comparative analysis of SRR8450805 & SRR8450806 from Zhang et al [2]

We obtained the latest release 1.01 of MAZTER-mine [3] from `https://github.com/SchwartzLab/mazter_mine`. After installing all necessary dependencies (samtools, bedtools and R), we followed the instructions under `https://github.com/SchwartzLab/mazter_mine/tree/master/tutorial`. Overall, It was easy to install and use. We selected the following individual samples and sorted them by read name:

```
#mock-treated RNA:
samtools sort -n -o SRR8450805_mazter_mine.bam SRR8450805_STARmapping_uniq_rmdup.bam
```

```
#FTO-treated RNA:
samtools sort -n -o SRR8450806_mazter_mine.bam SRR8450806_STARmapping_uniq_rmdup.bam
```

This step is followed by the extraction of read ends using 10 threads. This step (`bam2ReadEnds.R`) took **8 hrs per sample** on average using 10 CPU threads.

```
Rscript bam2ReadEnds.R -i SRR8450805_mazter_mine.bam -g final_annotation_96.bed -n 10

Rscript bam2ReadEnds.R -i SRR8450806_mazter_mine.bam -g final_annotation_96.bed -n 10
```

The next step, `mazter_mine.R`, computes a QC report and a cleavage efficiency table. This step took more than **10 hrs per sample** on average using 10 CPU threads.

```
Rscript mazter_mine.R -i SRR8450805_mazter_mine.Rdata -g final_annotation_96.bed \\
-f GRCh38_96.fa -u 60 -d 60 -n 10

Rscript mazter_mine.R -i SRR8450806_mazter_mine.Rdata -g final_annotation_96.bed \\
-f GRCh38_96.fa -u 60 -d 60 -n 10
```

In the manuscript of Garcia-Campos et al. [3], MAZTER-seq is used for validation of known sites and de novo discovery of m6A sites. However, the setting of Garcia-Campos et al. differs from the workflow of [2], which involves FTO treatment and which we use with JACUSA2. Garcia-Campos et al. suggest that an m6A-IP step is beneficial for the purpose of de novo detection of m6A sites and for quality control (QC)ing the performance of MAZTER-seq. As MAZTER-mine is mainly used for validation / quantitation of known sites, we decided to compare JACUSA2 and MAZTER-mine on the intersection of miCLIP defined m6A sites from [4, 5, 6] (intersect, see Figure S7). We only considered sites that have a coverage of 15 reads in both samples (SRR8450805 and SRR8450806), which is the default setting for MAZTER-mine. In summary, 88.7 % of all reported MAZTER-mine sites are also reported by JACUSA2. However, only 4 % and 28.3 % of all consensus miCLIP sites are reported by MAZTER-mine and JACUSA2 in this benchmark, respectively. The entire JACUSA2 analysis workflow took less than 30 min while MAZTER-mine took 18h (= 1080 min) per sample using the same hardware resources. Another draw back of MAZTER-mine is its dependency on gene annotation. JACUSA2 does not require gene annotation.

Benchmark: Nanopore m6A detection

We decided to do base calling from scratch for 2 samples, which were published in the xpore manuscript [7]: HEK293T-WT-0-rep1 and HEK293T-WT-100-rep1. Data were downloaded from `https://www.ebi.ac.uk/ena/browser/view/PRJEB40872`. While the latter is fully m6A modified (i.e. 100 % wildtype sample), the first is fully unmodified (i.e. KO situation). Briefly, we obtained the FAST5 files from EBI and performed standard guppy base calling on them (guppy version 5.0.11, rna_r9.4.1_70bps_hac model). The resulting FASTQ files (from the *pass* folder) were subsequently mapped with minimap2 (`-MD -ax splice -junc-bonus 1 -k14 -secondary=no`, version 2.22-r110) against the genome or transcriptome (EnsEMBL 96). Further details are given in each benchmark section below.

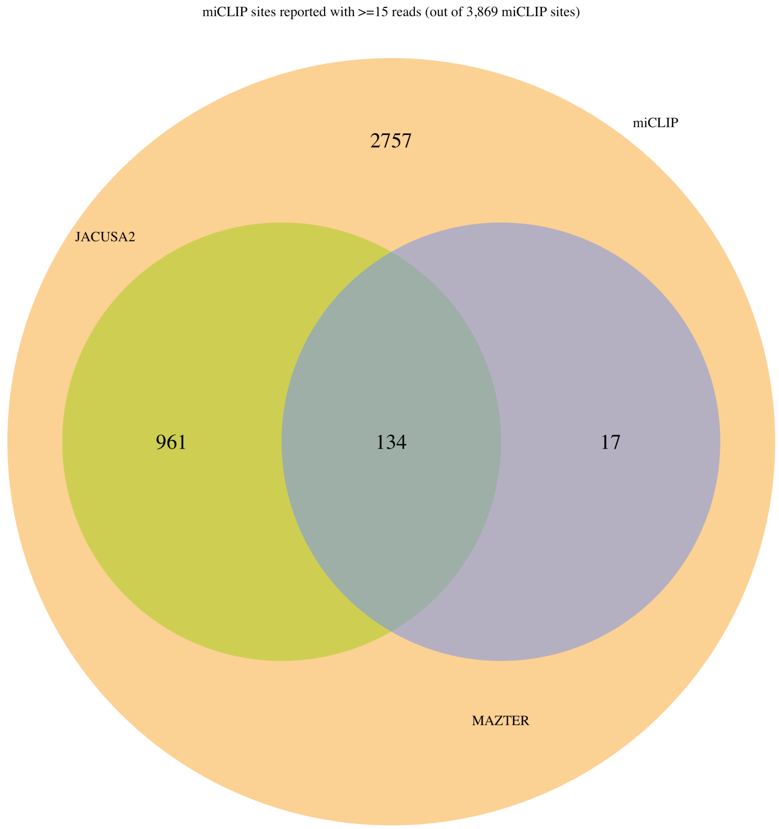

Figure S3: **Intersection of MAZTER-MINE and JACUSA2 with consensus HEK293 miCLIP data**

ELIGOS2

ELIGOS2 [8] is based on identifying characteristic base calling error profiles in the data. We obtained a singularity container of ELIGOS2 as explained below (Accessed on Jul 19 2021).

```
## Create Singularity image of ELIGOS2 from DockerHub
singularity build eligos2.sif docker://piroonj/eligos2:latest
```

After successful initialization, we performed all steps as outline under `https://gitlab.com/piroonj/eligos2#1-differential-esb-analysis-yeast-meiosis-transcriptome-to-identify-m6a-and-enriched-rrach-motif`. These analyses have been performed in a slurm compute cluster environment.

```
#preprocessing
srun -p long -c 10 singularity exec -e eligos2.sif eligos2 map_preprocess \\
-i guppy_called_HEK293T-WT-0-rep1.bam
srun -p long -c 10 singularity exec -e eligos2.sif eligos2 map_preprocess \\
-i guppy_called_HEK293T-WT-100-rep1.bam

# Run ELIGOS compare between samples when Wild-type (-tbam) and Knock-out (-cbam)
# using 10 CPU threads
srun -p long -c 10 singularity exec -e eligos2.sif eligos2 pair_diff_mod \\
-tbam guppy_called_HEK293T-WT-0-rep1.preprocess.bam \\
-cbam guppy_called_HEK293T-WT-100-rep1.preprocess.bam \\
```

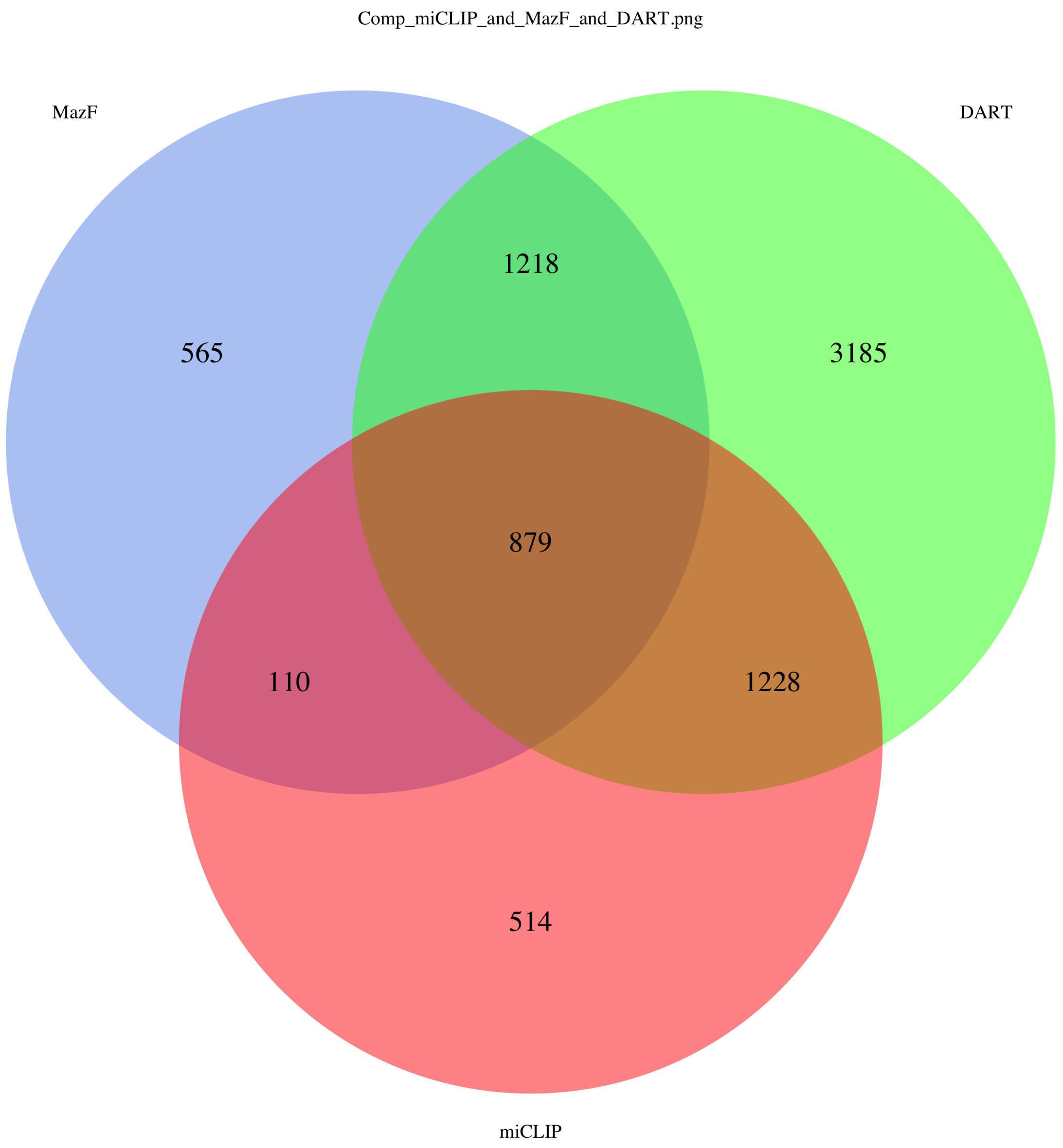

Figure S4: **Venn diagram of JACUSA2 predictions from MazF assay and DARTseq and overlap with miCLIP data.** miCLIP data is based on the intersection of 3 miCLIP experiments. Set numbers are computed on gene level. see also Additional File 2+3

```
-reg genes_hsa_96.bed -ref GRCh38_90.fa -t 10 --pval 0.001 \\
--oddR 1.2 --esb 0 -o WT100_vs_WT0_eligos2 --sub_bam_dir WT100_vs_WT0_temp

# Extract potential base A modified using eligos2 filter and filter out
# homopolymer sequence
srun -p long -c 10 singularity exec -e /home/cdieterich/software/eligos2.sif \\ eligos2 filter
-i guppy_called_HEK293T-WT-0-rep1.preprocess_vs_...preprocess_on_genes_hsa_96_baseExt0.txt \\
-sb A --homopolymer --esb 0 --oddR 1.2 --pval 0.001
```

Final output of ELIGOS2 consists of 1,907 m6A site predictions. However, only 41 ELIGOS2 predictions overlap with the union of three miCLIP experiments (72,815 sites). `ELIGOS2_intersect_with_miCLIP.bed`

The entire ELIGOS2 workflow, as shown above, took **16.8 days** (= 24,192 min), which is hardly conceivable for any practical use in the context of human or mammalian transcriptomes.

xpore

Xpore [7] is an ionic current/signal intensity based method. We installed `xpore/2.1` via pip install and as dependency `nanopolish/0.13.3`, which is required in the preprocessing steps. In our analysis, we basically follow the outlined steps as in `https://xpore.readthedocs.io/en/latest/quickstart.html`. The first pre-processing steps involve indexing of FAST5 files (`nanopolish index`), align to transcriptome using `minimap2` and Resquiggle using `nanopolish eventalign`. The `nanopolish eventalign` step was running on 10 CPU threads, but is time consuming (4-5 hours per Nanopore run). The actual xpore data preparation step (see below), which follows thereafter, took another 4-5 hours per sample.

```
# data preparation for the HEK293T-WT-0-rep1 sample
srun xpore dataprep --eventalign output_0/eventalign.txt --gtf_or_gff protein_coding.gtf \\
--transcript_fasta GRCh38_96_tx.fa --out_dir output_0_dataprep --genome
# data preparation for the HEK293T-WT-100-rep1 sample
srun xpore dataprep --eventalign output_100/eventalign.txt --gtf_or_gff protein_coding.gtf \\
--transcript_fasta GRCh38_96_tx.fa --out_dir output_100_dataprep --genome
```

Next step is modification calling and post-processing of results:

```
srun xpore diffmod --config Hek293T_config.yml --n_processes 10
srun xpore postprocessing --diffmod_dir finalOut
```

These last steps completed in $\approx$ 2.8 hours. Xpore outputs 81,121 candidate m6A sites for the human transcriptome. However, we had to derive genomic coordinates from EnsEMBL gene ids as the sequence identifier (i.e. chromosome) is not part of the output. In summary, 5,497 xpore predictions overlap with the union three miCLIP experiments (72,815 sites).

The entire Xpore workflow, as shown above, took **13 hrs** (= 780 min) per sample.

Epinano 1.2

Epinano [9] is based on identifying characteristic base calling error profiles in the data. We obtained a singularity container of ELIGOS2 as explained below (Accessed 20 Dec 2021).

```
#pull image from docker
singularity pull docker://huanleliu/epi12
```

Similar to JACUSA2, Xpore and ELIGOS2, we tried to analyze the complete data set. However, Epinano 1.2 did not complete. We suspect some issue related to Note 2 on README.md: the users should split the computations for each reference sequences if the reference genome is large.

```
#Does not work - hangs / forever
srun -p long -c 10 singularity exec -e epi12_latest.sif \\
python3 /usr/local/bin/EpiNano/Epinano_Variants.py \\
-R GRCh38_96.fa  -b .guppy_called_HEK293T-WT-0-rep1.bam \\
-s /usr/local/bin/EpiNano/misc/sam2tsv.jar -n 10 -T g
```

That is why, we reduced the data set to just include chromosome 1.

```
srun -p long -c 10 singularity exec -e epi12_latest.sif \\
python3 /usr/local/bin/EpiNano/Epinano_Variants.py -R GRCh38_96.fa  \\
-b guppy_called_HEK293T-WT-0-rep1-chr1.bam \\
-s /usr/local/bin/EpiNano/misc/sam2tsv.jar -n 10 -T g
srun -p long -c 10 singularity exec -e epi12_latest.sif \\
python3 /usr/local/bin/EpiNano/Epinano_Variants.py -R GRCh38_96.fa  \\
-b guppy_called_HEK293T-WT-100-rep1-chr1.bam \\
-s /usr/local/bin/EpiNano/misc/sam2tsv.jar -n 10 -T g
```

This step took already 3 hours just to complete on chromosome 1. We then tried to follow instructions as provided under `https://github.com/novoalab/EpiNano/blob/master/test_data/make_predictions/run.sh` using the docker container, but to no avail. We reckon that EpiNano 1.2 is not easy to install and to execute without substantial source code engineering and is unlikely to scale to larger data sets. In summary, we did not get any predictions from this workflow.

Comparison of ELIGOS2, Xpore and JACUSA2

The JACUSA2 workflow completes in less than 1 hour (60 minutes). JACUSA2 is orders of magnitude faster than competing solutions. JACUSA2 does not depend on and limits itself by gene annotation information. JACUSA2 enables m6A prediction outside of annotated regions and outputs genomic coordinates, which can be annotated repeatedly post hoc. There are 81,164 predictions from the xPore workflow as described above. Overlap with the union of miCLIP sites is 5,516 or 6.7 % of all xPore predictions. There are 1,907 predictions from the ELIGOS2 workflow as described above. Overlap with the union of miCLIP sites is 41 or 2.1 % of all ELIGOS2 predictions. Figure S5 summarizes the JACUSA2 predictions For the top 1,907 predictions from JACUSA2 (red dashed line), we observe an exact overlap of 15.4 % with all miCLIP sites (excluding the training sites). For the top 81,164 predictions from JACUSA2 (blue dashed line), we observe an exact overlap of 6.2 % with all miCLIP sites (excluding the training sites).

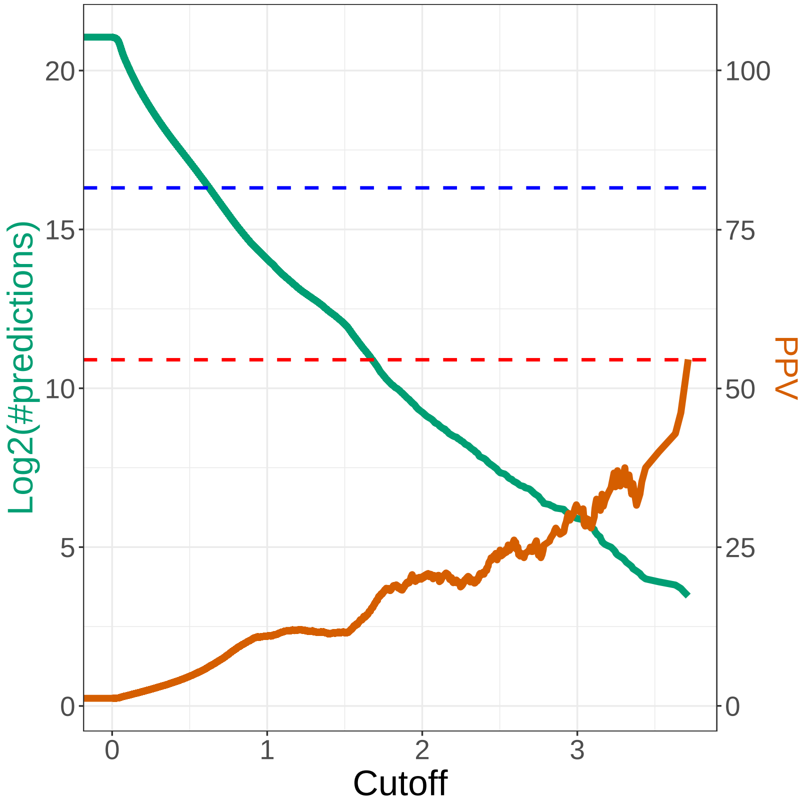

Figure S5: `JACUSA2` **predictions on human transcriptome** as represented in HEK293T-WT-0-rep1 and HEK293T-WT-100-rep1. Green line represents total number of predictions and brown line represents overlap with miCLIP data. Dashed lines represent ELIGOS2 (red) and xPore (blue) predictions respectively.

Knowledge transfer: m6A prediction in mouse embryonic stem cells

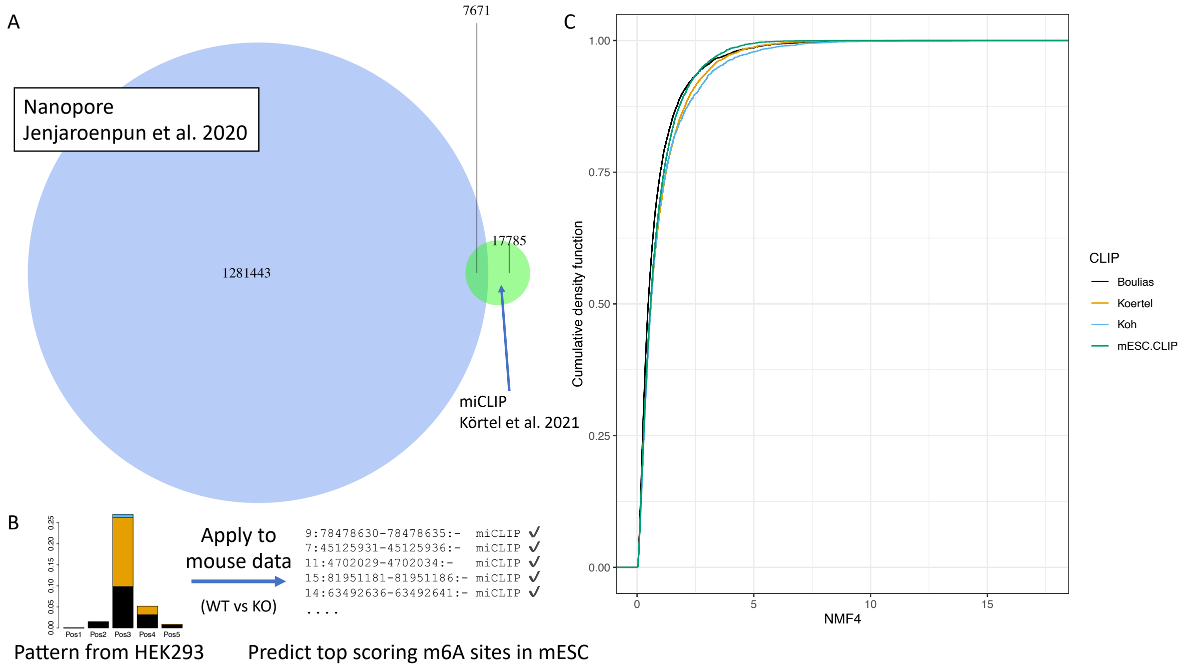

Figure S6: **Transfer of learned pattern from human to mouse** A: Characterization of 5mer-space between Nanopore direct RNA-seq data and miCLIP m6A sites from mouse embryonic stem cells. Overlap in terms of covered Nanopore 5mers is shown as intersection. B: Concept to carry over the pattern learned by NMF in HEK293 data to new data in a different species (i.e. mESCs). C: Cumulative score distribution are comparable for validated miCLIP sites in HEK293 or mESC systems.

Benchmark: Nanopore Ψ detection

We perform a benchmark for pseudouridine detection on human 18S rRNA. This part corresponds to the last section of the main text (Use Case 4: Nanopore direct rRNA sequencing). At the time of writing, a new software for Ψ detection was published: `nanopseudo_U` [10]. We retrieved the software from `https://github.com/sihaohuanguc/Nanopore_psU/releases/tag/v1.0`. It was easy to install and use. We basically follow the steps as outlined in `https://github.com/sihaohuanguc/Nanopore_psU#protocol`. The predictions approach uses 12 features (in comparison to 3 for JACUSA2). Please see original publication for details.

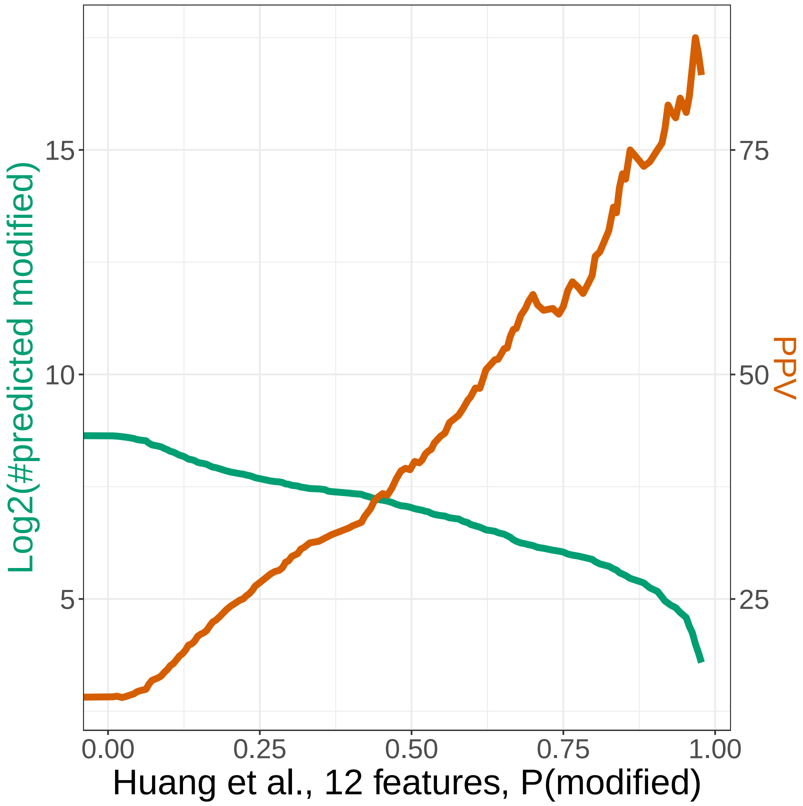

Figure S7: `nanopseudo_U` **predictions on human 18S rRNA** Briefly, 42 Ψ and 12 2'-O-methyl uridines are annotated as modified uridines along with 339 unmodified uridines.

Additional Use Case: Pseudouridylation site mapping in human rRNAs

Pseudouridylation (Ψ) is an abundant and widespread type of RNA epigenetic modification in living organisms. Specifically, rRNAs and tRNAs are well characterized targets of Pseudouridylation [11, 12]. In rRNA, pseudouridylation is carried out by ribonucleoprotein (RNP) complexes called H/ACA box RNPs, each consisting of one H/ACA snoRNA and four core proteins, namely GAR1, NHP2, NOP10 and dyskerin (DKC1) [13]. The recent identification of variable pseudouridylation sites has overturned the notion that all rRNA modifications are constitutively present on ribosomes, highlighting nucleotide modifications as an important source of ribosomal heterogeneity [14]. There are several ways to make Ψ sites accessible to sequencing-based profiling. Ψ sites have been mapped transcriptome-wide by chemically modifying pseudouridines with carbodiimide and detecting the resulting reverse transcription stops in high-throughput sequencing [15, 16, 17]. Specifically, Ψ sites on the human 80S rRNA are well annotated [11] and CMC-based rRNA profiling data from different cDNA libraries are available [15]. For our particular use case, we compiled a list of 104 Ψ^+ sites and 1,061 Ψ^- sites (i.e. other uridine residues), which are covered in all respective sequencing libraries (Figure S8A, Table S2). We consider read truncation, misincorporation, insertions and deletions from 3 experimental conditions as a 24-dimensional feature set to identify Ψ^+ sites (3 experiments $\times$ 4 feature $\times$ 2 positions (0,+1)).

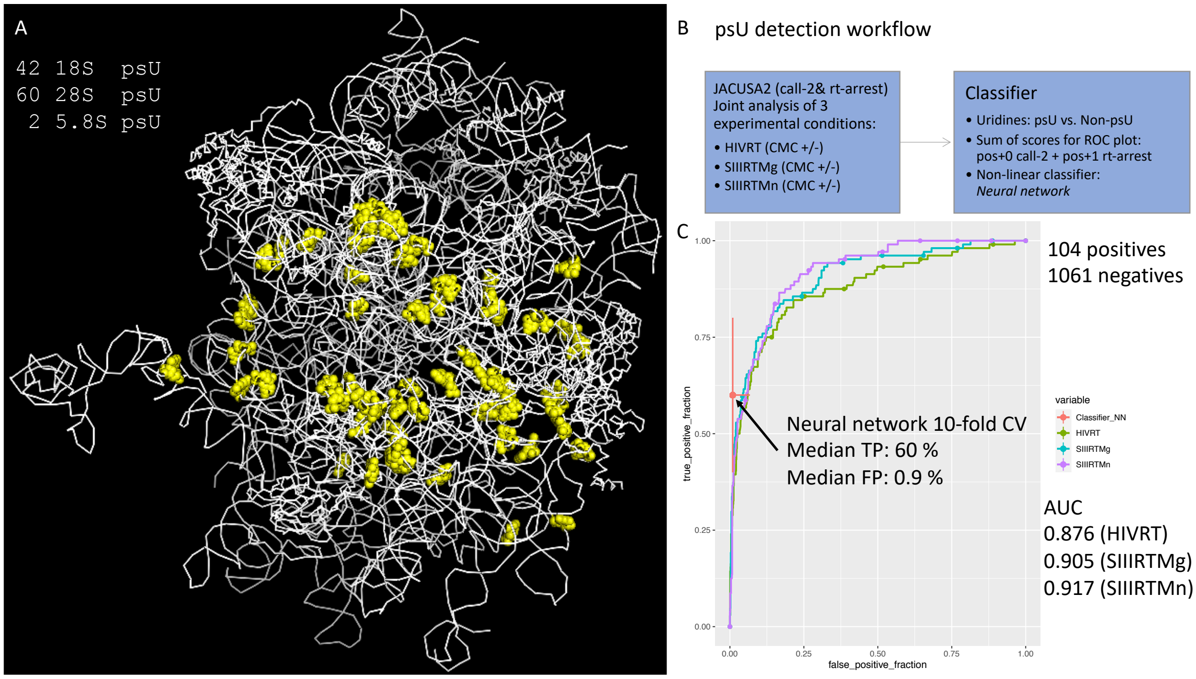

Figure S8: **Pseudouridine detection on 80S human ribosome using Illumina data from Zhou et al. [15]** A: Structure of the 80S ribosome (PDB file: `https://www.rcsb.org/structure/4UG0`) with Ψ positions highlighted in yellow. Annotation from [11]. B: Computational workflow to predict Ψ positions at all covered U positions. One simple predictor uses the sum of scores from rt-arrest at pos+1 and call-2 at pos+0 of different RTs and a Neural network classifier uses all x scores. C: ROC plot of simple scores colored by experimental condition. Neural network performance over a 10-fold cross-validation is depicted as cross-hair and median performance values are printed out.

Our computational workflow is depicted in Figure S8B. Briefly, we compute a simple score on individual experiments by summing up the call-2 score (base substitution) at every U (position 0) and the rt-arrest score (position +1). The corresponding ROC curve is plotted in Figure S8C. Our simple neural network classifier (1 input layer with 6 nodes, 1 hidden layer with 3 nodes, 2 output nodes) reaches a mean specificity of 99%

and a mean sensitivity of 60% sensitivity on hold-out test data.

For further details, please check the corresponding Vignette in https://github.com/dieterich-lab/JACUSA2helper

JACUSA2 parameters and downstream processing

Zhou et al. - Pseudouridines have context-dependent mutation and stop rates in high-throughput sequencing [15]

call-2 call

```
JACUSA2 Version: 2.0.0-RC22 call-2 -F 1024 -c 10 -p 10 -D -I -a D,Y,M -P1 FR-SECONDSTRAND \\
-P2 FR-SECONDSTRAND -r rmDup_SIIIRTMg_RC22_call2_result.out \\
rmDup/SIIIRTMg_PLUS_CMC_global_uniq\rmdup.bam rmDup/SIIIRTMg_MINUS_CMC_global_uniq_rmdup.bam
```

rt-arrest call

```
JACUSA2 Version: 2.0.0-RC22 rt-arrest -F 1024 -c 10 -p 10 -P1 FR-SECONDSTRAND \\
-P2 FR-SECONDSTRAND -r rmDup_SIIIRTMg_RC22_rtarrest_plain_result.out \\
rmDup/SIIIRTMg_PLUS_CMC_global_uniq_rmdup.bam \\
rmDup/SIIIRTMg_MINUS_CMC_global_uniq_rmdup.bam
```

Zhang et al. - Single-base mapping of m6A by an antibody-independent method [2]

rt-arrest call

```
JACUSA2 Version:  2.0.0-RC18 rt-arrest -F 1280 -c 4 -p 10 -P1 FR-SECONDSTRAND -P2 FR-SECONDSTRAND
-r MazF_vs_cond2_FTO_RC18_rtarrest_plain_result.out \\
SRR8450805_STARmapping_uniq_rmdup.bam,\\
SRR8450807_STARmapping_uniq_rmdup.bam,\\
SRR8450809_STARmapping_uniq_rmdup.bam\\
SRR8450806_STARmapping_uniq_rmdup.bam,\\
SRR8450808_STARmapping_uniq_rmdup.bam,\\
SRR8450810_STARmapping_uniq_rmdup.bam
```

Meyer - DART-seq: an antibody-free method for global m6A detection [18]

call-2 call

```
JACUSA2 Version:  2.0.0-RC18 call-2 -F 1024 -c 4 -p 10 -D -I -a D,Y -P1 RF-FIRSTSTRAND -P2
RF-FIRSTSTRAND -r APOBEC1YTH_APOBEC1YTHmut_RC18_call2_result.out \\
SRR9940470_STARmapping_uniq_rmdup.bam,\\
SRR9940471_STARmapping_uniq_rmdup.bam,\\
SRR9940472_STARmapping_uniq_rmdup.bam\\
SRR9940474_STARmapping_uniq_rmdup.bam,\\
SRR9940475_STARmapping_uniq_rmdup.bam,\\
SRR9940476_STARmapping_uniq_rmdup.bam
```

Pratanwanich et al. - Detection of differential RNA modifications from direct RNA sequencing of human cell lines [7]

```
JACUSA2 Version:  2.0.0-RC22 call-2 -m 1 -q 1 -c 4 -p 10 -D -I -a D,Y -P1 FR-SECONDSTRAND -P2
FR-SECONDSTRAND -r WT_vs_KO_2samp_RC22_call2_result.out \\
HEK293T-WT-rep2.bam,HEK293T-WT-rep3.bam \\
HEK293T-KO-rep2.bam,HEK293T-KO-rep3.bam
```

```
   JACUSA2 Version:  2.0.0-RC22 call-2 -m 1 -q 1 -c 4 -p 10 -D -I -a D,Y -P1 FR-SECONDSTRAND
-P2 FR-SECONDSTRAND -r WT100_vs_WT0_RC22_call2_result.out \\
HEK293T-WT-100-rep1.bam,HEK293T-WT-100-rep2.bam,HEK293T-WT-100-rep3.bam \\
HEK293T-WT-0-rep1.bam,HEK293T-WT-0-rep2.bam
```

Supplementary Table

Description of supplementary tables below.

Table S1: m6A predictions from MazF assay (FTO+/-) [2]

Microsoft XLSX file, 1.2MB, content: MazF.FTO - genomic coordinates, MazF.FTO - cDNA coordinates

Table S2: m6A predictions from DART-seq assay (YTH-domain) [18]

Microsoft XLSX file, 3.2MB, content: DART.genomic - genomic coordinates, DART.cDNA - cDNA coordinates

Table S3: m6A predictions from Nanopore (WT/KO) [7]

Microsoft XLSX file, 98MB, content: Nanopore.genomic - genomic coordinates, Nanopore.cDNA - cDNA coordinates

Table S4: Ψ predictions from Zhou et al. 2018 - HIV, SIII Mg + Mn / CMC + and -

Microsoft XLSX file, 70KB, column description:

```
ID - coordinates,
SIIIRTMnCall2Score_1 - Mismatch Score position 1 (U in reference),
SIIIRTMgCall2Score_1 - Mismatch Score position 1 (U in reference),
HIVRTCall2Score_1 - Mismatch Score position 1 (U in reference),
SIIIRTMnArrestScore_2 - Arrest Score position 2 (U+1 in reference),
SIIIRTMgArrestScore_2 - Arrest Score position 2 (U+1 in reference),
HIVRTArrestScore_2 - Arrest Score position 2 (U+1 in reference),
true label - as annotated in Taoka et al.  2018,
prediction - predicted label
```

Table S5: Ψ predictions from Nanopore (WT/IVT)

Microsoft XLSX file, 44KB, content:

```
ID - coordinates,
Exp1Call2Score_3 - Mismatch Score position 3 of 5mer (U in reference),
Exp1DeletionScore_3 - Deletion Score position 3 of 5mer (U in reference),
HIVRTInsertionScore_1 - Insertion Score position 3 of 5mer (U in reference),
true label - as annotated in Taoka et al.  2018,
Context - 5mer Context,
Level - modification level,
UMAP1,2 - projection coordinates,
SumOfScores - JACUSA2 final score
```

References

[1] Piechotta, M., Wyler, E., Ohler, U., Landthaler, M., Dieterich, C.: JACUSA: site-specific identification of RNA editing events from replicate sequencing data. BMC Bioinformatics **18**(1) (2017). doi:10.1186/s12859-016-1432-8

[2] Zhang, Z., Chen, L.-Q., Zhao, Y.-L., Yang, C.-G., Roundtree, I.A., Zhang, Z., Ren, J., Xie, W., He, C., Luo, G.-Z.: Single-base mapping of m6a by an antibody-independent method. Science advances **5**, 0250 (2019). doi:10.1126/sciadv.aax0250

[3] Garcia-Campos, M.A., Edelheit, S., Toth, U., Safra, M., Shachar, R., Viukov, S., Winkler, R., Nir, R., Lasman, L., Brandis, A., Hanna, J.H., Rossmanith, W., Schwartz, S.: Deciphering the "m6a code" via antibody-independent quantitative profiling. Cell **178**, 731–74716 (2019). doi:10.1016/j.cell.2019.06.013

[4] Boulias, K., Toczydłowska-Socha, D., Hawley, B.R., Liberman, N., Takashima, K., Zaccara, S., Guez, T., Vasseur, J.-J., Debart, F., Aravind, L., Jaffrey, S.R., Greer, E.L.: Identification of the m, javax.xml.bind.jaxbelement@3155288a, am methyltransferase pcif1 reveals the location and functions of m, javax.xml.bind.jaxbelement@72f29a24, am in the transcriptome. Molecular cell **75**, 631–6438 (2019). doi:10.1016/j.molcel.2019.06.006

[5] Körtel, N., Rücklé, C., Zhou, Y., Busch, A., Hoch-Kraft, P., Sutandy, F.X.R., Haase, J., Pradhan, M., Musheev, M., Ostareck, D., Ostareck-Lederer, A., Dieterich, C., Hüttelmaier, S., Niehrs, C., Rausch, O., Dominissini, D., König, J., Zarnack, K.: Deep and accurate detection of m6a rna modifications using miclip2 and m6aboost machine learning. Nucleic acids research **49**, 92 (2021). doi:10.1093/nar/gkab485

[6] Koh, C.W.Q., Goh, Y.T., Goh, W.S.S.: Atlas of quantitative single-base-resolution n6-methyl-adenine methylomes. Nature communications **10**, 5636 (2019). doi:10.1038/s41467-019-13561-z

[7] Pratanwanich, P.N., Yao, F., Chen, Y., Koh, C.W.Q., Wan, Y.K., Hendra, C., Poon, P., Goh, Y.T., Yap, P.M.L., Chooi, J.Y., Chng, W.J., Ng, S.B., Thiery, A., Goh, W.S.S., Göke, J.: Identification of differential rna modifications from nanopore direct rna sequencing with xpore. Nature biotechnology (2021). doi:10.1038/s41587-021-00949-w

[8] Jenjaroenpun, P., Wongsurawat, T., Wadley, T.D., Wassenaar, T.M., Liu, J., Dai, Q., Wanchai, V., Akel, N.S., Jamshidi-Parsian, A., Franco, A.T., Boysen, G., Jennings, M.L., Ussery, D.W., He, C., Nookaew, I.: Decoding the epitranscriptional landscape from native rna sequences. Nucleic acids research (2020). doi:10.1093/nar/gkaa620

[9] Liu, H., Begik, O., Lucas, M.C., Ramirez, J.M., Mason, C.E., Wiener, D., Schwartz, S., Mattick, J.S., Smith, M.A., Novoa, E.M.: Accurate detection of m6a rna modifications in native rna sequences. Nature communications **10**, 4079 (2019). doi:10.1038/s41467-019-11713-9

[10] Huang, S., Zhang, W., Katanski, C.D., Dersh, D., Dai, Q., Lolans, K., Yewdell, J., Eren, A.M., Pan, T.: Interferon inducible pseudouridine modification in human mrna by quantitative nanopore profiling. Genome biology **22**, 330 (2021). doi:10.1186/s13059-021-02557-y

[11] Taoka, M., Nobe, Y., Yamaki, Y., Sato, K., Ishikawa, H., Izumikawa, K., Yamauchi, Y., Hirota, K., Nakayama, H., Takahashi, N., Isobe, T.: Landscape of the complete rna chemical modifications in the human 80s ribosome. Nucleic acids research **46**, 9289–9298 (2018). doi:10.1093/nar/gky811

[12] Guzzi, N., Cieśla, M., Ngoc, P.C.T., Lang, S., Arora, S., Dimitriou, M., Pimková, K., Sommarin, M.N.E., Munita, R., Lubas, M., Lim, Y., Okuyama, K., Soneji, S., Karlsson, G., Hansson, J., Jönsson, G., Lund, A.H., Sigvardsson, M., Hellström-Lindberg, E., Hsieh, A.C., Bellodi, C.: Pseudouridylation of trna-derived fragments steers translational control in stem cells. Cell **173**, 1204–121626 (2018). doi:10.1016/j.cell.2018.03.008

[13] Penzo, M., Montanaro, L.: Turning uridines around: Role of rrna pseudouridylation in ribosome biogenesis and ribosomal function. Biomolecules **8** (2018). doi:10.3390/biom8020038

[14] Sloan, K.E., Warda, A.S., Sharma, S., Entian, K.-D., Lafontaine, D.L.J., Bohnsack, M.T.: Tuning the ribosome: The influence of rrna modification on eukaryotic ribosome biogenesis and function. RNA biology **14**, 1138–1152 (2017). doi:10.1080/15476286.2016.1259781

[15] Zhou, K.I., Clark, W.C., Pan, D.W., Eckwahl, M.J., Dai, Q., Pan, T.: Pseudouridines have context-dependent mutation and stop rates in high-throughput sequencing. RNA Biology **15**(7), 892–900 (2018). doi:10.1080/15476286.2018.1462654

[16] Carlile, T.M., Rojas-Duran, M.F., Zinshteyn, B., Shin, H., Bartoli, K.M., Gilbert, W.V.: Pseudouridine profiling reveals regulated mrna pseudouridylation in yeast and human cells. Nature **515**, 143–146 (2014). doi:10.1038/nature13802

[17] Schwartz, S., Bernstein, D.A., Mumbach, M.R., Jovanovic, M., Herbst, R.H., León-Ricardo, B.X., Engreitz, J.M., Guttman, M., Satija, R., Lander, E.S., Fink, G., Regev, A.: Transcriptome-wide mapping reveals widespread dynamic-regulated pseudouridylation of ncrna and mrna. Cell **159**, 148–162 (2014). doi:10.1016/j.cell.2014.08.028

[18] Meyer, K.D.: Dart-seq: an antibody-free method for global m6a detection. Nature methods **16**, 1275–1280 (2019). doi:10.1038/s41592-019-0570-0
