# Supplementary material for: RNA modification mapping with JACUSA2
Source: Genome Biol. 2022 May 16;23:115. doi: 10.1186/s13059-022-02676-0 (PMC9109409; doi:10.1186/s13059-022-02676-0)
Supplement: Supplementary file 7 — Additional file 7 Review history (DOCX). Peer review history. [file 13059_2022_2676_MOESM7_ESM.docx]

**1^st^ round**

**Reviewer 1**

In this study, the authors proposed an RNA modification mapping tool JACUSA2, with an additional R package JACUSA2helper for downstream analyses. To detect RNA modifications, JACUSA2 captures read signatures, which are base substitutions, insertions, deletions, and read truncations from BAM files of any read technology (Such as Illumina sequencing of MazF treatment libraries, DART-seq, and Nanopore direct RNA sequencing in the use cases). JACUSA2 is much faster than the authors' previous tool JACUSA. I downloaded and installed JACUSA2/JACUSA2helper from the provided GitHub URLs, and I was able to run the tools on my own data.

RNA modifications are essential during early development and are associated with disease. This work can be of broad interest in RNA modification research community. I have some concerns/suggestions, listed below.

Major

- page 3 line 15/16 and Figure 1G, in what cases a gene is judged to be modified? Is a gene is modified if there is at least one modified site in it?

- The authors apply JACUSA2 to extract read error profile differences and employ NMF to score the targeted sites from Nanopore data. However, in my opinion, the methodology and the assessment are unclear.

-- What does matrix V, W, H mean in Non-negative Matrix Factorization (in Figure 2C)? How does the NMF being trained and tested? More details should be given in the main text, in Figure 2C, or be added in supplementary.

-- page 3 line 59, maybe I miss it, but I can't understand where the "previously unseen miCLIP-predicted m6A sites" come from, with only Figure 2G presented.

-- In Figure 2H/Supplementary Figure 2C, there is no title of y-axis. What is the difference of NMF3 (in the legend) and NMF4 (in x-axis)?

- RNA modification detection using Nanopore data are under active development now. Besides methods like JACUSA2 which are base-calling and alignment-based, there are also ionic current/signal intensity based methods [1]. If possible, I suggest that the authors compare JACUSA2 with other base-calling and alignment-based methods (like ELIGOS [2] for m6A detection), and the ionic current/signal intensity based methods, like xPore [3] for m6A detection and NanoRMS [4] for pseudouridine detection, to strengthen their work. The comparison may be shown as Figure 1G (overlap of predicted modified bases) and Supplementary Figure 3C (ROCAUC).

References

[1] Furlan M, Delgado-Tejedor A, Mulroney L, et al. Computational methods for RNA modification detection from nanopore direct RNA sequencing data[J]. RNA biology, 2021: 1-10.

[2] Jenjaroenpun P, Wongsurawat T, Wadley T D, et al. Decoding the epitranscriptional landscape from native RNA sequences[J]. Nucleic Acids Research, 2021, 49(2): e7-e7.

[3] Pratanwanich P N, Yao F, Chen Y, et al. Identification of differential RNA modifications from nanopore direct RNA sequencing with xPore[J]. Nature Biotechnology, 2021: 1-9.

[4] Begik O, Lucas M C, Pryszcz L P, et al. Quantitative profiling of pseudouridylation dynamics in native RNAs with nanopore sequencing[J]. Nature Biotechnology, 2021: 1-14.

- page 4 line 42/Figure 3A, according to what I see, I don't think the dots in different colors in Figure 3A are well separated. I also don't understand what Um, m1asp3psU, and psU is, respectively. The authors should explain more about this figure.

Minor

- page 1 line 29, I don't think "Reverse transcription signature" and "Multi-threading" should be included in Keywords, as they don't appear either in Abstract or in the main text.

- I suggest that the authors spell out or explain the acronyms the first time they are used. For example, page 1 line 45, JACUSA; page 2 line 46, FTO.

- page 1 line 59, what does "citation eligos2" mean?

- page 3 line 55/56, "most relevant pattern (Figure 2D)", is it "Figure 2E" which should be mentioned here?

- page 5 line 6-10, please check if STab 1 and STab 2 are mentioned in the right places.

**Reviewer 2**

This paper reported JACUSA2, an updated version of JACUSA, a software developed previously for m6A and other mRNA modification site detection from RNA modification sequencing protocols. This updated software package is specifically designed to detect m6A and other RNA modifications from antibody-free sequencing data, including restriction enzyme-based such as MaZF, fusion-domain methods like DART-seq, and nanopore sequencing data. All core performance assessments were performed with HEK293 cell line data. The authors demonstrated improvements in detection coverage and computing resources over their previous versions of this software. Comparisons of JACUSA2 detection results from different sequencing protocols on HEK293 cell line data were performed. However, the utility of this software could be limited because of the oversimplification of the algorithm and a lack of systematic comparison with existing approaches.

Major concerns:

1. An attractive feature of JACUSA2 is that it could be applied to data from different sequencing protocols. The authors claim that JACUSA2 takes bam files directly as the input and outputs modification sites. However, the trade-off of this universal applicability is that its algorithm failed to consider protocol-specific challenges. For instance, for MazF based protocols including MASTER-seq and m6A-REF-seq, many factors including two close-by ACA sequences and the presence of G neighboring an ACA sequence could impact ACA cleavage efficiency and therefore detection accuracy of the algorithm. For nanopore sequencing, pore current signals due to RNA modification could be highly complex and require careful analysis of error signal features or training of machine learning algorithms with high confidence grand truth modification sites (as in Eligos, Epinano, or MINES). The proposed algorithm does not consider any of those protocol-specific features. Although the paper "trained" an NMF for nanopore sequencing data, the training data is formed rather ad hoc.

2. What is also missing is rigorous comparisons of JACUSA2 with existing detection approaches for respective sequencing protocols (MASTER-Mine for Mater-seq and Epinano etc for nanopore, or at least comparisons with reported detected sites from the existing papers.) As a result, the extent to which one might favor JACUSA2 over specialized methods for different sequencing data could not be assessed.

Minor concerns:

1. Page 1, convert "cite eligo2" to citation.

2. The author should clarify the specific nanopore data that were used.

3. In page 4, line 9, sites were supported by more than one miCLIP experiments showed higher scores than ONT data. But in Figure 2H, scores of the Boulias dataset are the highest and those overlapped with three datasets (Boulias, Koertel, Kor) seem have the lowest scores. There is a discrepancy that this needs clarification.

4. Figure 2H and Supplementary Figure 2C, the x-axes of both figures are NMF4, but the figure caption says NMF3. Please check the consistency.

5. Figure 1E: The legend/caption/ and descriptions are confusing.

**Reviewer 3**

This manuscript reports the successor of JACUSA, which was published in BMC Bioinformatics (2017). That prior publication describes the statistical model used for calling SNV in RNA-RNA and DNA-RNA comparisons. This new package purports "better running time performance and captures more complex read signatures involving base substitutions, insertions, deletions, and read truncations". Overall, I think this would be a welcome addition to the tools available for detection of RNA modifications by "antibody-free methods to map RNA modifications transcriptome-wide based on high-throughput sequencing data", but the use cases as presented are confusing and need substantial clarification.

I think that the following issues should be addressed:

1. Please reference the Supplementary Material earlier in the text, as it contains vital information about the Methods that are critical for evaluating this new package (including details of the new rt-arrest analysis mode, and documentation of run time improvements). These aspects of the Supplementary Material referenced at all; I suggest adding a sentence to clarify that this material is available, probably in the second paragraph of the "Background" (note that Genome Biology short reports should contain no headings anyway).

2. For each "use case", please be explicit about the JACUSA mode being used (call-1, call-2, pileup, etc.). This is shown in the Supplementary Material but should be highlighted in the text.

3. The details of Fig 1 panels D, E, F, and H are too small to clearly convey information at 100% magnification. Please revise this panel to enlarge these panels or at least the panel text. Perhaps the Figure legend could extend onto an adjacent page.

4. Fig 1D took a while to figure out; please be more explicit in describing what is going on here with the four samples. Part of the problem is that the panel is too small to clearly see the plot lines and text. Fig 1E: text notes that arrest rates are significantly higher in FTO samples but no statistical value for the comparison is given. Please provide the method and statistical test result for the comparison data shown.

5. Fig 1F-1H: This analysis seems to be using the variant calling module, which was available in the original JACUSA. How does the JACUSA2 analysis differ from the prior tool, if at all? The use case demonstrates that the tool works (JACUSA2), but presumably it also worked before (JACUSA)? It would be helpful to highlight what is new/better here in the text. Maybe it is just faster?

6. Fig 1F: Please explain exactly what is being plotted here. Figure legend for 1F implies 17k points from DART-seq and FTO-MazF, but axes only represent YTH binding domains, and the text only refers to the Meyer data. Points along the line of equality (X=Y) seem to have been suppressed.

7. Fig 1H: what is the Y-axis? Fn(x) = cumulative probability?

8. Fig 2C and D: is the unsupervised learning (NMF) applied within JACUSA2? How this is achieved in the JACUSA2 modes described is unclear from the text and supplementary material. Panel 2E is not referenced in the text at all. In 2H, the y-axis is simply labeled as y; I think this should be cumulative probability. Also in 2H, the x-axis is labeled as NMF4 (but the legend references NMF3), with markings 0-20. 0-20 of what?

**Reviewer 4**

In the report, the authors propose an improved version JACUSA2 of their previous JACUSA software for RNA modification data processing. The new features include shorter running time and more complex read signatures, such as substitutions, insertions, and deletions. Several use cases demonstrate the versatility of JACUSA2 on analyzing data from antibody-free, genetic, or nano pore sequencing approaches. The following suggestions may be considered to further improve the manuscript.

1. Comparisons to other existing methods are needed, although the authors' own JACUSA (previous versions) are compared. The methods should be evaluated on several benchmarks and a summary of their pros and cons are recommended.

2. In page 3 and Figure 1, can the authors use the "intersection" of the three miCLIP datasets and compare it to JACUSA2? The "intersection" should have a more reliable sets of genes and modified sites than the "union".

3. In Figure 1H, can a p-value to reported to see whether the difference between MazF and DART is significant?

4. I would suggest to show a couple of examples of JACUSA2helper as the downstream analyses of JACUSA2.

5. The rRNA sequencing (Naarmann-de Vries et al. in preparation) information should be made available to the reader to better understand the application of JACUSA2 on this dataset.

6. The fonts of some subfigures are too small to be seen and should be enlarged

**Authors’ response**

Reviewer #1: In this study, the authors proposed an RNA modification mapping tool JACUSA2, with an additional R package JACUSA2helper for downstream analyses. To detect RNA modifications, JACUSA2 captures read signatures, which are base substitutions, insertions, deletions, and read truncations from BAM files of any read technology (Such as Illumina sequencing of MazF treatment libraries, DART-seq, and Nanopore direct RNA sequencing in the use cases). JACUSA2 is much faster than the authors' previous tool JACUSA. I downloaded and installed JACUSA2/JACUSA2helper from the provided GitHub URLs, and I was able to run the tools on my own data. RNA modifications are essential during early development and are associated with disease. This work can be of broad interest in RNA modification research community. I have some concerns/suggestions, listed below.

>> We would like to thank the reviewer for this valuable assessment.

Major

- page 3 line 15/16 and Figure 1G, in what cases a gene is judged to be modified? Is a gene is modified if there is at least one modified site in it?

>> Yes, the interpretation of the reviewer is correct. A gene is classified as modified if at least one site is modified within.

However, we have improved on the presentation of results and appearance of Figure 1.

Briefly, old Figure 1A-C was moved to supplement (now Sfig 1 in Supplementary Text). Old Figure 1D and E are now enlarged for better visibility.

New Figure 1C-E have been redesigned to show the overlap with the intersect of 3 miCLIP data sets depending on JACUSA p-value (maZF) or score (DARTseq). Figure 1E (old Figure 1H) offers larger labels for better readability.

The Venn diagram has been moved to Supplementary Text (Sfig 3).

- The authors apply JACUSA2 to extract read error profile differences and employ NMF to score the targeted sites from Nanopore data. However, in my opinion, the methodology and the assessment are unclear.

-- What does matrix V, W, H mean in Non-negative Matrix Factorization (in Figure 2C)? How does the NMF being trained and tested? More details should be given in the main text, in Figure 2C, or be added in supplementary.

>>We compose a matrix V with dimensions n and m over every considered 5mer (NNANN). Herein, n is the number of considered 5mer positions and m=30 (i.e. 3 features: mismatch, insertion, deletion over 5 positions over 2 experiments). This matrix is decomposed into an Amplitude matrix W with dimensions n*rank and into a Pattern Matrix with dimensions rank*30. This aspect has been clarified in the main text.

-- page 3 line 59, maybe I miss it, but I can't understand where the "previously unseen miCLIP-predicted m6A sites" come from, with only Figure 2G presented.

>> We have adjusted Figure 2G accordingly. The "3-overlap" was used as training data (red colored now) wheras all other columns in Figure 2G are use as test data (shown in light blue).

-- In Figure 2H/Supplementary Figure 2C, there is no title of y-axis. What is the difference of NMF3 (in the legend) and NMF4 (in x-axis)?

>>We replaced Figure 2H with a plot showing the number of JACUSA2 predictions and perfect overlap with miCLIP sites (test data) based on the profile score as shown in Figure 2E. The overlap is defined as positive predicitive value for miCLIP sites: #TP / (#TP + #FP).

Supplementary Figure 2C is now 6C and was corrected.

- RNA modification detection using Nanopore data are under active development now. Besides methods like JACUSA2 which are base-calling and alignment-based, there are also ionic current/signal intensity based methods [1]. If possible, I suggest that the authors compare JACUSA2 with other base-calling and alignment-based methods (like ELIGOS [2] for m6A detection), and the ionic current/signal intensity based methods, like xPore [3] for m6A detection and NanoRMS [4] for pseudouridine detection, to strengthen their work. The comparison may be shown as Figure 1G (overlap of predicted modified bases) and Supplementary Figure 3C (ROCAUC).

>> We have tried to follow all reviewer's suggestions. However, we made an important observation in this regard. Neither ELIGOS2 nor Epinano 1.2 (which we also considered due to another reviewer's request) offered result tables for mammalian transcriptome m6A sites predictions. To the contrary, xPore does for HEK293 data. In fact, we use the xPore data set here. We ammended Figure 2H with dashed lines. These represent the xPore performance as published in Supplementary Table 1 of xPore paper.

In Supplementary text page 6ff, we explained how we setup a benchmark from scratch. However, much to our dismay, it was impossible for us to install and execute the Epinano 1.2 workflow. Moreover, ELIGOS2 had a prohibitively high run time of 16 days, which does not seem practical in comparison to xPore and JACUSA2. Please take a look at Supplementary Text page 6 - Benchmark: Nanopore m6A detection. For pseudouridine modifications, we decided to benchmark against a recent software solution, nanopseudo\_U, which we found easy to install and execute. See page 11 of Supplementary Text.

\#

References

[1] Furlan M, Delgado-Tejedor A, Mulroney L, et al. Computational methods for RNA modification detection from nanopore direct RNA sequencing data[J]. RNA biology, 2021: 1-10.

[2] Jenjaroenpun P, Wongsurawat T, Wadley T D, et al. Decoding the epitranscriptional landscape from native RNA sequences[J]. Nucleic Acids Research, 2021, 49(2): e7-e7.

[3] Pratanwanich P N, Yao F, Chen Y, et al. Identification of differential RNA modifications from nanopore direct RNA sequencing with xPore[J]. Nature Biotechnology, 2021: 1-9.

[4] Begik O, Lucas M C, Pryszcz L P, et al. Quantitative profiling of pseudouridylation dynamics in native RNAs with nanopore sequencing[J]. Nature Biotechnology, 2021: 1- 14.

- page 4 line 42/Figure 3A, according to what I see, I don't think the dots in different colors in Figure 3A are well separated. I also don't understand what Um, m1asp3psU, and psU is, respectively. The authors should explain more about this figure.

>> Indeed, reviewer's obervation indicates that uridine modifications as represented by pseudouridine, 2-0-Methylated uridine and a single complex modification do not separate well from one another.

However, new Figure 3B quantifies JACUSA2 ability to separate modified uridines from unmodified uridines. Moreover, JACUSA performs almost en par with Nanopore_psU, which is tailored solution for pseudouridine detection.

In this comparison, we simply used 3 features: mismatch, insertion and deletion at each 18S rRNA uridine position, while Nanopore_psU uses 12 features (see original pubication).

Minor

- page 1 line 29, I don't think "Reverse transcription signature" and "Multi-threading" should be included in Keywords, as they don't appear either in Abstract or in the main text.

>> We added an explanation of "Reverse transcription signature" to the main text and removed Multi-threading" from the list of keywords.

- I suggest that the authors spell out or explain the acronyms the first time they are used. For example, page 1 line 45, JACUSA; page 2 line 46, FTO.

>> Thank you very much for pinpointing this issue to us. We have corrected this matter throughout our manuscript.

- page 1 line 59, what does "citation eligos2" mean?

>> Replaced it with a proper reference citation.

- page 3 line 55/56, "most relevant pattern (Figure 2D)", is it "Figure 2E" which should be mentioned here?

>> Indeed, we have corrected this reference.

- page 5 line 6-10, please check if STab 1 and STab 2 are mentioned in the right places.

>> We have replaced this part with a general reference to supplementary Tables.

Reviewer #2: This paper reported JACUSA2, an updated version of JACUSA, a software developed previously for m6A and other mRNA modification site detection from RNA modification sequencing protocols. This updated software package is specifically designed to detect m6A and other RNA modifications from antibody-free sequencing data, including restriction enzyme-based such as MaZF, fusion-domain methods like DART-seq, and nanopore sequencing data. All core performance assessments were performed with HEK293 cell line data. The authors demonstrated improvements in detection coverage and computing resources over their previous versions of this software. Comparisons of JACUSA2 detection results from different sequencing protocols on HEK293 cell line data were performed. However, the utility of this software could be limited because of the oversimplification of the algorithm and a lack of systematic comparison with existing approaches.

>> We would like to thank the reviewer for these valuable comments and provide a comparison to exisiting approaches. For details see below.

Major concerns:

1.An attractive feature of JACUSA2 is that it could be applied to data from different sequencing protocols. The authors claim that JACUSA2 takes bam files directly as the input and outputs modification sites. However, the trade-off of this universal applicability is that its algorithm failed to consider protocol-specific challenges. For instance, for MazF based protocols including MASTER-seq and m6A-REF-seq, many factors including two close-by ACA sequences and the presence of G neighboring an ACA sequence could impact ACA cleavage efficiency and therefore detection accuracy of the algorithm. For nanopore sequencing, pore current signals due to RNA modification could be highly complex and require careful analysis of error signal features or training of machine learning algorithms with high confidence grand truth modification sites (as in Eligos, Epinano, or MINES). The proposed algorithm does not consider any of those protocol-specific features. Although the paper "trained" an NMF for nanopore sequencing data, the training data is formed rather ad hoc.

>> Indeed, JACUSA2 could be seen as a swiss-army knife for RNA modification research. We do not expect it to perform better than highly specialized solutions for each and every use case.

A key advantage of JACUSA2 is its easy deployment, flexibility and speed. JAVA software runs anytime, anywhere and does not require any sophisticated installation. JACUSA2 is complemented with an R helper package, which eases downsteam processing of JACUSA2 output. We are certain that this software will be highly valuable for many end users who would like to repeatedly experiment with different settings and features in RNA modification analyses.

<<

2.What is also missing is rigorous comparisons of JACUSA2 with existing detection approaches for respective sequencing protocols (MASTER-Mine for Mater-seq and Epinano etc for nanopore, or at least comparisons with reported detected sites from the existing papers.) As a result, the extent to which one might favor JACUSA2 over specialized methods for different sequencing data could not be assessed.

>>Indeed, we tried to benchmark JACUSA2 vs a number of published software solutions (see also response to reviewer #1: MAZTER-Mine for Illumina data, ELIGOS2, Epinano 1.2 and xPore 2.1 for m6A detection on the Nanopore and Nanopore_psU for pseudouridine detection on Nanopore data. MAZTER-seq is used for validation / quantitation of known sites and de novo discovery of m6A sites provided that m6A IP data are available. So, we decided to compare our predictions with a consensus miCLIP data set. see new Figure 1 C&D and see Supplementary Material - Benchmarking JACUSA2 against other published software - page 6 and SFig 3.

We have tried to follow all reviewer's suggestions. However, we made an important observation in this regard. Neither ELIGOS2 (which we also considered) nor Epinano 1.2 offered result tables for mammalian transcriptome m6A sites predictions. To the contrary, xPore does for HEK293 data. In fact, we use the xPore data set here. We ammended Figure 2H with dashed lines. These represent the xPore performance as published in Supplementary Table 1 of xPore paper.

In Supplementary Material page 6ff, we explained how we setup a benchmark from scratch. However, much to our dismay, it was impossible for us to install and execute the Epinano 1.2 workflow. Moreover, ELIGOS2 had a prohibitively high run time of 16 days, which does not seem practical in comparison to xPore and JACUSA2. Since, we observed a poor performance, we decided to only include our observations as part of the supplementary materials (see section Benchmark: Nanopore m6A detection) <<

Minor concerns:

1.Page 1, convert "cite eligo2" to citation.

Thank you for notifying us. We correctly cite the reference now.

2.The author should clarify the specific nanopore data that were used.

This information was added to supplementary text - see Pratanwanich et al. - Detection

of differential RNA modifications from direct RNA sequencing of human cell lines

3.In page 4, line 9, sites were supported by more than one miCLIP experiments showed higher scores than ONT data. But in Figure 2H, scores of the Boulias dataset are the highest and those overlapped with three datasets (Boulias, Koertel, Kor) seem have the lowest scores. There is a discrepancy that this needs clarification.

We replaced Figure 2H with an easier interpretable one.

4.Figure 2H and Supplementary Figure 2C, the x-axes of both figures are NMF4, but the figure caption says NMF3. Please check the consistency.

Corrected.

5.Figure 1E: The legend/caption/ and descriptions are confusing.

We tried to improve in the caption and main text.

Reviewer #3: Genome Biology

GBIO-D-21-01611

RNA modification mapping with JACUSA2

This manuscript reports the successor of JACUSA, which was published in BMC Bioinformatics (2017). That prior publication describes the statistical model used for calling SNV in RNA-RNA and DNA-RNA comparisons. This new package purports "better running time performance and captures more complex read signatures involving base substitutions, insertions, deletions, and read truncations". Overall, I think this would be a welcome addition to the tools available for detection of RNA modifications by "antibody-free methods to map RNA modifications transcriptome-wide based on high-throughput sequencing data", but the use cases as presented are confusing and need substantial clarification.

I think that the following issues should be addressed:

1.Please reference the Supplementary Material earlier in the text, as it contains vital information about the Methods that are critical for evaluating this new package (including details of the new rt-arrest analysis mode, and documentation of run time improvements). These aspects of the Supplementary Material referenced at all; I suggest adding a sentence to clarify that this material is available, probably in the second paragraph of the "Background" (note that Genome Biology short reports should contain no headings anyway).

>> Thank you very much for pointing out this important aspect. We have added "Our manuscript is accompanied by Supplementary Material (see Additional File 1), which provides detailed information on methods, benchmarks and performance." to the last paragraph of the Background section.

Please note that this part has been expanded considerably.

2.For each "use case", please be explicit about the JACUSA mode being used (call-1, call-2, pileup, etc.). This is shown in the Supplementary Material but should be highlighted in the text.

We have added the corresponding information to the main text.

3.The details of Fig 1 panels D, E, F, and H are too small to clearly convey information at 100% magnification. Please revise this panel to enlarge these panels or at least the panel text. Perhaps the Figure legend could extend onto an adjacent page.

We have newly designed Figure 1. Old panels A+B+C were moved to the supplement. Please take a look.

4.Fig 1D took a while to figure out; please be more explicit in describing what is going on here with the four samples. Part of the problem is that the panel is too small to clearly see the plot lines and text. Fig 1E: text notes that arrest rates are significantly higher in FTO samples but no statistical value for the comparison is given. Please provide the method and statistical test result for the comparison data shown.

We have enlarged the respective panels (now 1A+B) and added statistical test results (see Figure 1 caption).

5.Fig 1F-1H: This analysis seems to be using the variant calling module, which was available in the original JACUSA. How does the JACUSA2 analysis differ from the prior tool, if at all? The use case demonstrates that the tool works (JACUSA2), but presumably it also worked before (JACUSA)? It would be helpful to highlight what is new/better here in the text. Maybe it is just faster?

The DARTseq analysis used indeed the conventional call-2 module. However, the current call-2 module is enhanced with insertion and deletion events and runs considerably faster (see Supplementary Text - SFig 2).

6.Fig 1F: Please explain exactly what is being plotted here. Figure legend for 1F implies 17k points from DART-seq and FTO-MazF, but axes only represent YTH binding domains, and the text only refers to the Meyer data. Points along the line of equality (X=Y) seem to have been suppressed.

We have moved Figure 1F to the vignette of the JACUSA2helper package see https://dieterich-lab.github.io/JACUSA2helper/articles/JACUSA2helper- figure1f.html

Indeed, only dots with a JACUSA2 score >= 2 are shown. New Figure 1D shows the overlap with miCLIP sites based on scores.

7.Fig 1H: what is the Y-axis? Fn(x) = cumulative probability?

We have replaced Figure 1H with new Figure 1E.

8.Fig 2C and D: is the unsupervised learning (NMF) applied within JACUSA2? How this is achieved in the JACUSA2 modes described is unclear from the text and supplementary material. Panel 2E is not referenced in the text at all. In 2H, the y-axis is simply labeled as y; I think this should be cumulative probability. Also in 2H, the x-axis is labeled as NMF4 (but the legend references NMF3), with markings 0-20. 0-20 of what?
We agree with the reviewer that this part was a bit confusing. We have redesigned Figure 2E and edited the corresponding section in the main text plus figure caption for clarity.

Reviewer #4: In the report, the authors propose an improved version JACUSA2 of their previous JACUSA software for RNA modification data processing. The new features include shorter running time and more complex read signatures, such as substitutions, insertions, and deletions. Several use cases demonstrate the versatility of JACUSA2 on analyzing data from antibody-free, genetic, or nano pore sequencing approaches.

The following suggestions may be considered to further improve the manuscript

>> We would like to thank the reviewer for the submitted constructive assessment.

1. Comparisons to other existing methods are needed, although the authors' own JACUSA (previous versions) are compared. The methods should be evaluated on several benchmarks and a summary of their pros and cons are recommended.

>> Indeed, we spent a considerable amount of time to benchmark JACUSA2 against other software solutions: MAZTER-MINE, Epinano 1.2, ELIGOS2 and xPore. Our observation was (see comments to reviewer #1-3) that JACUSA2 is orders of magnitude faster, is easy to install and use, does not require and is not limited by genome annotation and performs equally well or better than other software. See Figure 1 +2 and extensive Supplementary Text for details.

2. In page 3 and Figure 1, can the authors use the "intersection" of the three miCLIP datasets and compare it to JACUSA2? The "intersection" should have a more reliable sets of genes and modified sites than the "union".

>> We have replaced the union with the intersection in new Figure 1C+D and in new Supplementary Figure 3.

3. In Figure 1H, can a p-value to reported to see whether the difference between MazF and DART is significant?

We have replaced Figure 1H with new Figure 1E for better readability and added a p- value from wilcoxon rank sum test to figure caption.

4. I would suggest to show a couple of examples of JACUSA2helper as the downstream analyses of JACUSA2.

Thank you very much for your suggestion. However, due to space limitations we are forced to refer the reader to

<https://dieterich-lab.github.io/JACUSA2helper/index.html>

5. The rRNA sequencing (Naarmann-de Vries et al. in preparation) information should be made available to the reader to better understand the application of JACUSA2 on this dataset.

We have updated the availability of data and material section. The novel rRNA sequencing data was used in use case 4 and corresponding benchmark, but will be made available upon publication.

6. The fonts of some subfigures are too small to be seen and should be enlarged

We tried to improve wherever possible and hope to meet the expectations of the reviewer.

**2^nd^ round**

**Reviewer 3**

Overall, the Authors have sufficiently addressed my original concerns. I will note that the revised text still suffers somewhat from a lack of clarity in how the evidence is presented to support JACUSA2’s utility in “calling” m6A sites, but this is somewhat due to the general uncertainty about the precise identification of m6A sites with various experimental methods (and therefore no “gold standard” for reference). Perhaps this limitation is seen best in Fig 3, where the 18S rRNA pseudouridines are well-defined, and the PPV when detecting a majority of sites (say 2^5=32) is only 25%. Despite this, I still think that the JACUSA2 package will be a useful tool for mapping RNA modifications. Note that, given the extensive revisions, a few minor issues remain:

Case 1: I suggest clarifying that arrest rate = cleavage efficiency (which is the terminology used by MATZER-seq publication)

Fig 1E: Assay annotation (DART vs MazF) is much too small to read

Fig 2F is not referenced in the text.

Fig 3: Since there are only 54 modified uridines on 18S rRNA, please use a linear Y-axis instead of log2(#predicted modified). It will make it clearer how well the scoring system correctly calls these sites.

On Supplement page 11, “Benchmark Nanopore Y detection”, there is an unreferenced reference: “This part corresponds to the last section of the main text (REF).”

**Authors’ response**

Reviewer #3: Overall, the Authors have sufficiently addressed my original concerns. I will note that the revised text still suffers somewhat from a lack of clarity in how the evidence is presented to support JACUSA2's utility in "calling" m6A sites, but this is somewhat due to the general uncertainty about the precise identification of m6A sites with various experimental methods (and therefore no "gold standard" for reference). Perhaps this limitation is seen best in Fig 3, where the 18S rRNA pseudouridines are well-defined, and the PPV when detecting a majority of sites (say 2^5=32) is only 25%.

Despite this, I still think that the JACUSA2 package will be a useful tool for mapping RNA modifications. Note that, given the extensive revisions, a few minor issues remain:

Authors ́ Response: We thank the reviewer for his careful assessment and appreciation of JACUSA2.

Case 1: I suggest clarifying that arrest rate = cleavage efficiency (which is the terminology used by MATZER-seq publication)

We added one sentence to the respective section:

Briefly, 3 replicates were treated with an Alpha-ketoglutarate-dependent dioxygenase (FTO), which converts m6A to A on mRNA [14], and 3 replicates were mock-treated.

MazF cleavage efficiency (or arrest rate) does increase in the absence of m6A.

Fig 1E: Assay annotation (DART vs MazF) is much too small to read

Fixed

Fig 2F is not referenced in the text.

It is now included in the main text.

Fig 3: Since there are only 54 modified uridines on 18S rRNA, please use a linear Y- axis instead of log2(#predicted modified). It will make it clearer how well the scoring system correctly calls these sites.

We think there is a misunderstanding here. In the main text we state:

"Briefly, 42 Ψ and 12 2’-O-methyl uridines have been mapped on human 18S rRNA along with 339 unmodified uridines." So, we consider 42+12+339 positions in total.

On Supplement page 11, "Benchmark Nanopore Y detection", there is an unreferenced reference: "This part corresponds to the last section of the main text (REF)."

Fixed
